# Isomers of Iron(III) Oxides and Cobalt(III) Oxides and Their Redox Properties: Quantum-Chemical Insights

**DOI:** 10.3390/molecules30214158

**Published:** 2025-10-22

**Authors:** Sapajan Ibragimov, Leonard Komando, Maciej Bobrowski

**Affiliations:** 1 Faculty of Chemistry, Gdańsk University of Technology, Narutowicza 11/12, 80-222 Gdańsk, Poland; sapajan.ibragimov@pg.edu.pl; 2 Department of Exact Science, Khorezm Mamun Academy, Markaz-1, Khiva 220900, Uzbekistan; 3 Faculty of Technical Physics and Applied Mathematics, Gdańsk University of Technology, Narutowicza 11/12, 80-222 Gdańsk, Poland; leonard.komando@pg.edu.pl

**Keywords:** iron oxide, cobalt oxide, redox potential, quantum chemistry, electron affinity

## Abstract

Iron(III) oxide and cobalt(III) oxide can form distinct spatial and spin configurations. Kite, spindle, and linear geometries have been shown to be stable for the specified electron configurations; however, these oxides generally favor higher open-shell configurations, which are ferromagnetic or antiferromagnetic. Reduction and oxidation reactions affect the geometry and spin states of these systems, sometimes leading to isomer transformations. Calculated standard reduction potentials of iron trioxides against the Standard Hydrogen Electrode (SHE) range from −0.37 V to −0.72 V, depending notably on the oxide geometry, spin, and computational method employed. For cobalt trioxides, standard reduction potentials range from −0.63 V to +0.18 V. Ionization potentials range from approximately 8 eV to 10 eV for iron oxides and from about 9 eV to 10 eV for cobalt oxides. Electron affinity values range from 2.36 eV to 2.76 eV for iron oxides and from 2.47 eV to about 2.94 eV for cobalt oxides, with these values being more sensitive to the computational method employed and the specific isomer considered. Consequently, iron(III) and cobalt(III) oxides are about three times more susceptible to one-electron reduction than oxidation. Specifically, kite-shaped Fe_2_O_3_ and Co_2_O_3_ configurations are most vulnerable to reduction. Conversely, the linear configuration of iron oxide and cobalt oxide exhibits the lowest susceptibility to oxidation, as indicated by their elevated ionization potentials. Overall, both iron(III) and cobalt(III) oxides act as relatively effective redox agents.

## 1. Introduction

Iron(III) oxide, i.e., iron trioxide or ferric oxide, is a very popular compound. It can be formed naturally or by employing many different methods. One of the most common processes is the rusting of iron. In the laboratory, it can be obtained by the electrolysis of aqueous sodium chloride or baking soda, using an iron anode submerged in the solution. In these conditions, the anode undergoes oxidation to iron(III) ions. The intermediate, hydrated iron(III) oxide, i.e., rust, also written as Fe(O)OH, undergoes dehydration at elevated temperatures and converts to red brown ferric oxide. The anodizing of iron and steel is not beneficial because rust is porous and hygroscopic and is not effective in preventing additional corrosion, in comparison to, for instance, the anodizing of aluminum or titanium [1]. Despite this, highly ordered iron oxide nanotubes with smooth and homogeneous walls were synthesized by potentiostatic anodization of iron foil in ethylene glycol [2]. Also, good-quality iron trioxide films were achieved via the LPCVD and PVD processes to protect iron from corrosion [3,4]. Actually, to obtain red brown ferric oxide, one needs to perform dehydration at elevated temperatures. Also, it is well known that stronger oxidizers or even oxygen molecules can change iron (II) salts or their hydroxides into ferric hydroxides, but the process of forming ferric oxide must also include dehydration at elevated temperatures. When it comes to nano- and microparticles, quite a few different methods have actually been developed: physical methods, chemical methods, and microbiological ones [5,6,7,8]. Among them, the most popular chemical pathway to obtain nanoparticles is the wet (co)precipitation method [6,9]. With this method, typically ferrous and/or ferric salts in an aqueous medium under a controlled pH are employed, while magnetite can subsequently undergo oxidation to iron trioxide. Actually, the mechanism is not simple. Specifically, in water, iron cations, both ferrous and ferric, form hexacoordinated aqua complexes, and the process of forming oxides from such complexes involves many intermediate compounds. Typically, in the first stage, the process involves increasing the pH and the precipitation of hydroxides, which have more extensive structures, in the form of aqua hydroxo and oxo complexes. In the next steps, the complexes undergo condensation. There are two distinct mechanisms of this process, but both involve a substitution mechanism, induced by the nucleophilic character of a hydroxo or oxo ligand. These mechanisms are called olation and oxolation and involve the formation of hydroxo and oxo bridges, releasing water molecules [10,11,12]. Frequently, the formation of amorphous ferrihydrite primary particles or prenucleation clusters has been reported in numerous previous studies as an intermediate phase in iron oxide formation [10,13,14,15]. So far, there have been no detailed reports on the specific chemical pathways leading from the precursors to nanostructures. However, a thorough molecular understanding of the chemical basis of phase separation is crucial for tailoring the size, shape, and structure of novel nanomaterials or more crystalline forms – including the determination of their key properties [12]. Regardless of this fact, wet chemical synthesis remains a relatively simple and energy-saving routine in nanoparticle preparation. Several other methods have been reported in the literature, including microemulsions, sonochemical reactions, electrochemical deposition, hydrothermal reactions, flow injection synthesis, electrospray, and flame synthesis [16,17]. However, some of them also belong to the group of wet synthesis, where the precursors are iron salts or alkoxides. Also, the relation between the properties of small molecules and those of nanoparticles and crystals is not known. In the so-called green approach, plants, bacteria, fungi, and algae are used to fabricate iron oxide nanoparticles, resulting in varied forms and sizes falling in the 1 to 100 nm range [7]. In these methods, iron precursors are hydrated iron salts or iron complexes, which next undergo chemical reactions along biochemical pathways. In this regard, the cell wall and soluble enzymes are involved in the synthesis, while the chemical mechanisms are not known in detail. The advantage is that the temperature is not as high as in chemical or physical methods. During the synthesis, positively charged metal ions can aggregate on the surface of microbial cells because most microorganisms possess a negative surface charge due to the presence of anionic carboxyl and phosphate groups [18]. It was also proven that ferric oxide displays antibacterial properties [19]. Many of these methods allow for achieving well-defined, versatile shapes and controlled sizes of the particles.

Iron oxides play an important role in geology, biology, medicine, and industry and are of great interest [20,21]. It is well known that iron oxides can display good magnetic properties, which are strongly correlated with the high spin and the crystalline order of the materials. Nevertheless, they can also exhibit redox properties, which are most likely related to the presence of an iron(III) ion. The redox activity can also probably influence the magnetic properties, because it is related to the change in the electronic structure. Sun et al. synthesized electrodes coated with Fe_2_O_3_ nanoparticles and used them in batteries [22]. As reported, the new Fe_2_O_3_ based electrode, used with 1-ethyl-3-methylimidazolium tetrafluoroborate (EMIMBF_4_) ionic liquid (acting as the electrolyte), was able to operate fully reversibly in a voltage range of 0 to 4 V and exhibited a very high energy density of 177 Wh kg^−1^. As demonstrated, the pseudocapacitive behavior of Fe_2_O_3_ in EMIMBF_4_ was closely related to the chemical state variation between Fe^3+^ and Fe^2+^ on the surface of the Fe_2_O_3_ nanoparticle layer [22]. The electrochemical properties of α Fe_2_O_3_ (hematite) films were also shown in the application of photoanodes for water splitting [23]. Cobalt oxides were generally synthesized using wet and biological methods with various precursors (salts and alkoxides) [24,25]. a Cobalt oxides have been reported to possess significant antioxidant, antibacterial, and cytotoxic activities [25]. It is obvious that these properties result from the oxidative properties of the Co(III) ion present in their structure. Cobalt complexes are well known as active redox couples [26,27,28,29,30].

Nanoscale transition metal oxide clusters have been extensively studied due to their unique properties, which differ significantly from those of their bulk counterparts. These metal oxide clusters are known to form various isomers and exhibit distinct reactivity, even at the same cluster size. They play a crucial role as catalysts and catalytic supports in a wide range of applications [31], including the hydrogen evolution reaction [32,33,34]. A key area of interest in metal oxide clusters is their ability to facilitate redox reactions. The study of small metal oxides can lead to a deeper understanding of the physical and chemical properties of larger catalytic systems.

To further analyze the redox attributes, one must first obtain reliable configurations of the compounds for both the reduced and oxidized states. Furthermore, because transition metals and their ions can display various spin states, and because the spins generally differ in the presence of different ligands and ions surrounding the metals (in the case of the oxides, the oxygen atom can be referred to as the oxo ion (O^2−^)), it is necessary to determine the relationships between the spatial and spin configurations for all reliable combinations. Several studies have attempted to find the local minima of iron trioxides, even though they were not necessarily related to the chemical pathways involved in the synthesis of oxide materials. Erlebach et al. used the classical Born–Mayer potential along with the density functional PBE exchange-correlation functional. In their work, all DFT computations for the bulk structures were performed using periodic boundary conditions and the VASP package [35]. Even though the authors constructed only certain highly symmetric spatial configurations (for instance, for the smallest iron trioxide, they considered only the kite-like structure) and arbitrarily imposed selected spins, they claimed to have found the global minima. The authors postulated that the lowest-energy state of iron trioxide was the C_2*v*_ kite-like configuration with total spin S = 0 (singlet state). In an earlier paper by Erlebach et al. [36], in a similar manner, only very limited types of structures of (Fe_2_O_3_)_n_, n = 1 to 5, were taken into consideration. Despite this, the authors also claimed to have found the global minimum structures of neutral (Fe_2_O_3_)_*n*_ clusters with n = 1–5. Ding et al. used the B3LYP method along with LANL2DZ (in most cases), as well as 6-311+G(d) basis sets, and found many minimum energy cage and noncage structures of iron trioxides [37]. The authors showed that the cage structures were stable, but the noncage structures were the global minima in most cases. The authors did not investigate the smallest iron trioxide structures. The classical empirical potential energy function method was applied for various metal (M) oxides (O) with the general formula (MO)_*n*_, (M_3_O_4_)_*n*_, (M_2_O_3_)_*n*_, (MO_2_)_*n*_, (M_2_O_5_)_*n*_, (MO_3_)_*n*_, for *n* ranging from 1 to 60, depending on the stoichiometric formula [38]. The authors did not consider the spin of the molecules, and all structures were highly symmetric. Moreover, for each size of the oxide, only one type of spatial configuration was taken into consideration. Ultimately, their conclusion was that according to their method, oxides could form only highly symmetric compact structures. In the case of the smallest M_2_O_3_ particle, the spindle-like structure was found to be the most stable, even though no other configurations were considered. For M_4_O_6_, the adamantane-like structure was identified as the lowest energy configuration. In all the above cases, even the type of metal element was not defined, and the cases of, for instance, cobalt oxides and iron oxides were not even distinguished. Of course, it is impossible to estimate the electronic redox properties using classical potential methods. Also, for electronic structure methods, it is important to know the spin of the system. This is even more important considering that low-spin iron and cobalt oxides can exhibit antiferromagnetic properties. Therefore, it remains important to investigate the properties of the lowest-energy oxides, even if they do not necessarily reliably represent the states at characteristic points along their specific synthesis reaction paths, because there may be a specific function connecting the properties of nanocompounds and the properties of small, reliable systems. This was the motivation for our work, beginning with the smallest possible states of iron(III) and cobalt(III) oxides.

As already cited, iron(III) and cobalt(III) oxide systems, including nanosystems, are known to possess good redox properties. Cobalt(III) salts and complexes exhibit superior redox properties compared to the corresponding iron(III) systems. Metal oxide systems differ significantly from salts and complexes; they can be stoichiometrically “repeatable” and assume large sizes, configurations, and shapes, being quite flexible in this respect. In the periodic table, cobalt and iron atoms are neighbors and have high spins. Since metal oxides differ markedly from the salts of these metals or their complexes, we aimed to investigate how their redox reactions proceed in iron and cobalt oxides and how these properties relate to the redox properties of salts and complexes. By elucidating the detailed structure of iron(III) and cobalt(III) oxide systems—starting from the smallest possible structures, taking into account their spin, and calculating their redox and related properties for stable configurations—we can determine the relationship between the properties of oxides and compare them with those of salts and complexes. There appears to be a functional relationship between the properties of such systems, especially since oxides can contain a very large number of metal atoms. The electronic structure of both salts and complexes, as well as oxides, directly affects the redox properties (high spin, presence of non-bonding orbitals, and large number of π orbitals). It is also necessary to identify truly stable states of isomers, conducting research in an analytical manner and, in the case of theoretical calculations, validating the methods used, as larger molecular systems will not be included in some calculations. Only theoretical studies can combine information about the structure, size, shape, oxidation state, type of metal atom, doping with other metals, and redox potentials in a systematic and analytical manner. Similar studies have been performed, among others, for copper complexes [39], but in the case of metal oxides, the diversity of their structure and spin also requires a theoretical approach to find a function that combines all these properties in the future.

## 2. Results and Discussion

### 2.1. Validation of the Methods: The Case of the FeO Molecule

First, we validated the methods by assessing the accuracy of MP2 and DFT with the aug−cc−pVTZ basis set (a Dunning-type cc-contracted basis set augmented with extensive polarization and diffuse functions—up to g-type for iron and up to f-type for oxygen—and one set of diffuse functions). We optimized the various spin states of the FeO molecule and its anion FeO− to identify the lowest-energy states; both methods yielded the same result (see Tables S13 and S14 in the Supplementary Material), namely that the lowest-energy states of the FeO molecule and its anion are, respectively, the quintet and the sextet [40,41], consistent with the CASPT2 ((12 and 13), 14) computations and slow photoelectron spectroscopy measurements [40]. Next, we calculated the adiabatic electron affinity, as shown in Table 1.

FeO has an electron affinity of 1.4950 eV [40]. The CASPT2/ANO-RCC computations (ANO-R basis sets are all-electron basis sets developed at the CASPT2 level and augmented with a set of diffuse primitive functions [42]) provided an adiabatic electron affinity of the FeO molecule equal to 1.52 eV [41]. The RB3LYP/aug−cc−pVTZ computations yielded an electron affinity of 1.22 eV, while the RMP2/aug−cc−pVTZ computations resulted in a value of 1.52 eV. In the case of the FeO molecule, the unrestricted approximation gives slightly lower EA and IP values. The problem with CASSCF computations is that they become very expensive as the CAS size increases; even for the small FeO molecule, the CAS consisted of 14 orbitals. For Fe_2_O_3_, the number of CAS orbitals would likely be much larger, practically excluding such computations. Moreover, iron and cobalt trioxide molecules are the smallest possible stoichiometric representatives of larger systems, including nanoparticles, making the configuration interaction approach impractical. Therefore, one must seek cheaper yet efficient methods. In the case of the FeO molecule, an antiferromagnetic configuration does not occur, and therefore, this effect did not need to be considered or compared with external data. The DFT with the B3LYP functional method was recently validated for computing the redox properties of polyoxometalates [43], demonstrating good agreement with experimental redox potentials. In some cases, the MP2 method fails [44]. However, for iron (II) oxide, this method produces significantly better results than the DFT method.

### 2.2. Iron and Cobalt Trioxides’ Geometry and Spin Configurations

**Figure 1:**
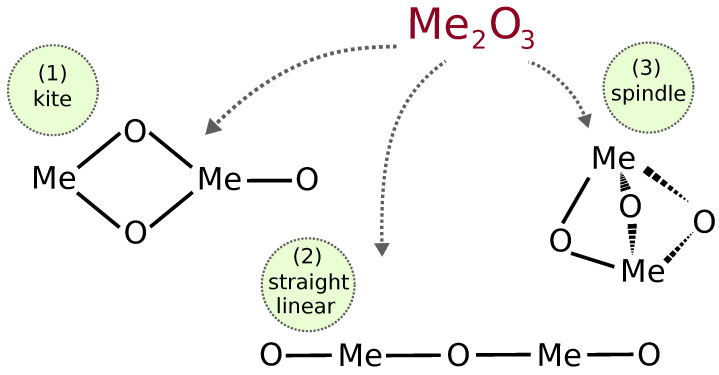
Depiction of 3 possible alternating geometries of metal trioxides: Fe_2_O_3_ and Co_2_O_3_.

We started our studies by manually constructing geometries, maintaining stoichiometry, and imposing appropriate combinations of charges and spins. We build the following types of geometries, where iron or cobalt atoms alternate in connecting to oxygen atoms: kite-like, spindle-like, and linear configurations (see Figure 1). The spindle structure is the tightest and most compact; the kite structure can be formed from the spindle geometry by breaking one of the Me–O bonds, and the linear configuration can be generated by breaking an additional Me–O bond of the spindle.

To thoroughly investigate the formation of appropriate electronic states for specific spatial isomers using two completely different methods, it was necessary to consider the possibility of including the antiferromagnetic configuration. In fact, taking into account the antiferromagnetic and ferromagnetic configuration (for spin 0—nonmagnetic), we obtain a set of two different states for each isomer. We first present the complete results, including antiferromagnetic states obtained at the DFT level with the aug−cc−pVTZ basis set, followed by an analysis of the ferromagnetic and antiferromagnetic states obtained using the MP2 method with the same basis set.

#### 2.2.1. UB3LYP/aug−cc−pVTZ Results

**Figure 2:**
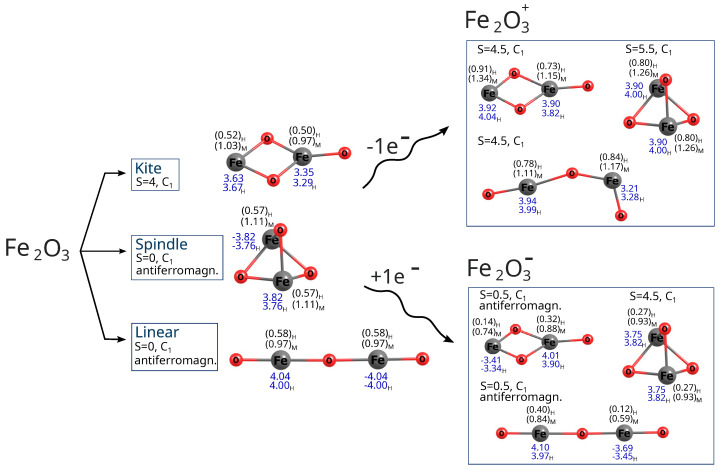
Molecular structures of UB3LYP/aug−cc−pVTZ that optimized the lowest energy states of iron trioxides and the corresponding cations and anions. Total spins (S) were shown for each optimized state. No symmetry was imposed on the geometry. The structures were optimized in vacuum. Mulliken (M) and Hirshfeld (H) partial charges were shown in parentheses for the iron atom. Spin density values are shown in blue. If a given lowest-energy state among all those considered was characterized by an antiferromagnetic arrangement of spins, then this was marked in the drawing.

**Figure 3:**
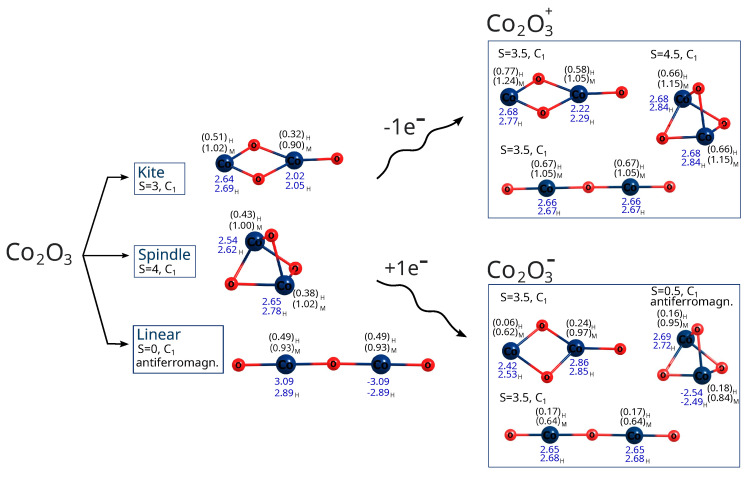
Molecular structures of UB3LYP/aug−cc−pVTZ that optimized the lowest energy states of cobalt trioxides and the corresponding cations and anions. Total spins (S) were shown for each optimized state. No symmetry was imposed on the geometry. The structures were optimized in a vacuum. Mulliken (M) and Hirshfeld (H) partial charges are shown in parentheses for the cobalt atom. Spin density values are shown in blue. If a given lowest-energy state among all those considered was characterized by an antiferromagnetic arrangement of spins, then this is marked in the drawing.

**Figure 4:**
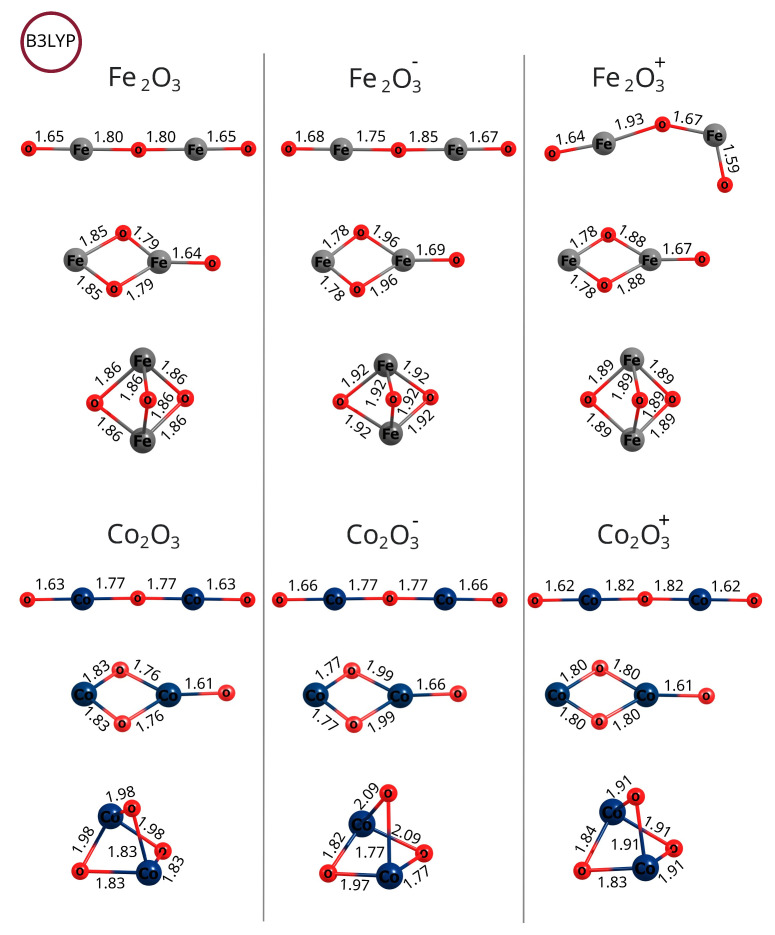
Bond lengths (Å) of the lowest-energy configurations of iron(III) and cobalt(III) oxides found at the UB3LYP/aug−cc−pVTZ level of theory.

**Figure 5:**
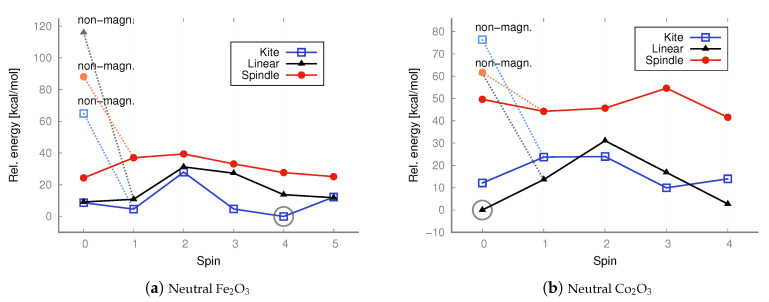
UB3LYP/aug−cc−pVTZ relative energies [kcal/mol] calculated with respect to the lowest-energy state of kite iron trioxide (S = 4) and linear cobalt trioxide (S = 0; antiferromagnetic conf.), respectively. The energies of both singlet states with non-magnetic (non-magn.) and antiferromagnetic configurations were taken into account. The lowest energy is marked with a circle.

**Figure 6:**
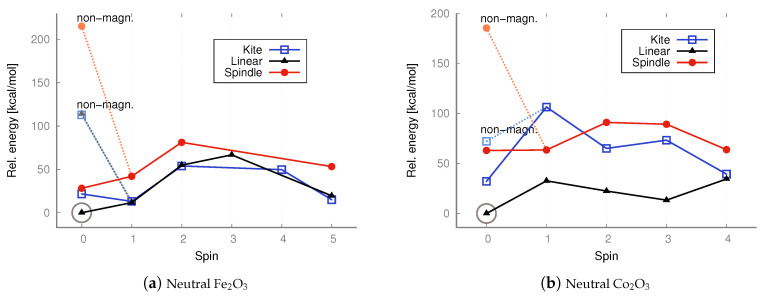
UMP2/aug−cc−pVTZ relative energies [kcal/mol] calculated with respect to the lowest-energy anti-ferromagnetic singlet (S = 0) state of the linear iron and cobalt trioxide, respectively. Non-magnetic singlet states are marked in a lighter color. The lowest energy is marked with a circle.

The molecular structures of the UB3LYP/aug−cc−pVTZ–optimized local minima of the lowest-energy states are shown in Figure 2 and Figure 3. The geometric parameters are presented in greater detail in Figure 4. Both nonmagnetic and antiferromagnetic singlet states were considered. Not all spin states of neutral molecules were achievable, depending also on the methods employed. For instance, with the DFT method, all states (for all spins considered) of iron and cobalt trioxides were stable, whereas with the MP2 method, spindle configurations of both oxides were achievable only for certain imposed spins. For cobalt oxide, this limitation also applied to the linear configuration (see Figure 5 and Figure 6). Moreover, not all geometry types showed stable cationic and anionic states. After a one-electron reduction or oxidation reaction, neutral isomers often experienced the breaking of one or two Me–O bonds, transforming into different isomers depending on the system spin. Some isomers also changed geometries upon changing spin state or after redox reactions. The initially linear molecule of iron trioxide became bent, and after one-electron oxidation followed by geometry optimization, it became further bent (see Figure 2).

Since many states of Fe_2_O_3_ and Co_2_O_3_ contain unpaired electrons in their outermost shells, and due to their generally high-spin or antiferromagnetic nature, we chose to perform all computations using spin-unrestricted Hartree–Fock (UHF) wave functions. Furthermore, the DODS approximation allows one to naturally obtain an antiferromagnetic state, corresponding by definition to the singlet open-shell state. Although UHF wave functions are not eigenfunctions of the spin-squared operator, ${\hat{S}}^{2}$, they can formally be expanded in terms of pure spin states of higher multiplicities. UHF eigenfunctions have a considerable advantage over restricted open-shell HF (R(O)HF) wave functions, particularly in accurately representing bond dissociation energies and spin densities in open-shell systems. Therefore, we applied MP2 and DFT methods associated with UHF reference wave functions, in which case the unperturbed Hamiltonian and Kohn–Sham operators are given by sums of the spin-up (α) and spin-down (β) Fock operators, reflecting spin contamination. However, the ground-state structures of Fe_2_O_3_^(0,+1,−1)^ and Co_2_O_3_^(0,+1,−1)^ observed with the UMP2 and UB3LYP methods exhibit modest spin contamination, as shown in Tables S5–S8. The largest spin contamination was observed for antiferromagnetic configurations. Since spin contamination could lead to false conclusions in specific cases, we performed additional calculations using restricted Kohn–Sham orbitals and the BHHLYP exchange-correlation functional, recently validated in computing redox properties [43]. The states obtained by RBHHLYP are not spin-contaminated but cannot describe antiferromagnetic configurations. Thus, we obtained all plausible ferromagnetic and non-magnetic states for The three isomers of iron trioxides and compared their energies (Figure S3). The restricted BHHLYP/aug−cc−pVTZ energy charts as a function of spin clearly indicate that iron trioxide isomers prefer higher spins, while low-spin states, including the nonmagnetic (S = 0) state, have significantly higher energies. Similar conclusions arise from unrestricted DFT and MP2 computations (detailed below), with the antiferromagnetic singlet state having lower energy than nonmagnetic and even some ferromagnetic states.

**Figure 7:**
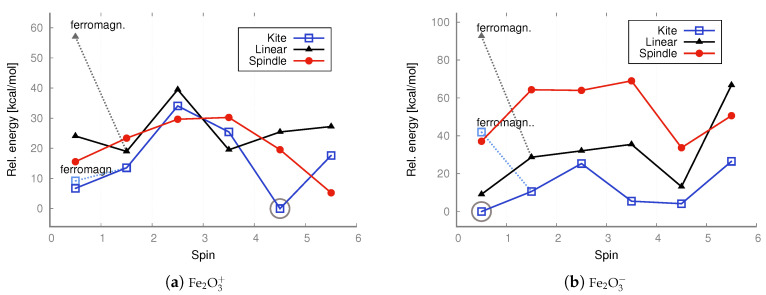
DFT UB3LYP/aug−cc−pVTZ relative energies [kcal/mol] calculated with respect to the lowest-energy state of ferromagnetic cation (S = 4.5) or anion (S = 1/2, including antiferromagnetic conf.) of a kite iron trioxide, respectively. The energies of both doublet states—with ferromagnetic (ferromagn.) and antiferromagnetic configurations—were taken into account. The lowest energy is marked with a circle.

**Figure 8:**
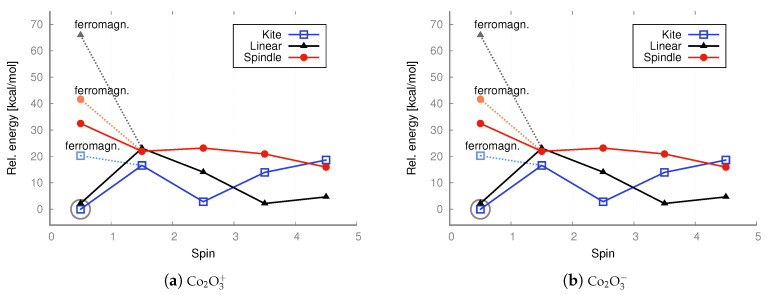
DFT UB3LYP/aug−cc−pVTZ relative energies [kcal/mol] calculated with respect to the lowest-energy doublet state (S = 1/2, including antiferromagnetic conf.) of the cation and anion of a kite cobalt trioxide, respectively. The energies of both doublet states—with ferromagnetic (ferromagn.) and antiferromagnetic configurations—were taken into account. The lowest energy is marked with a circle.

**Figure 9:**
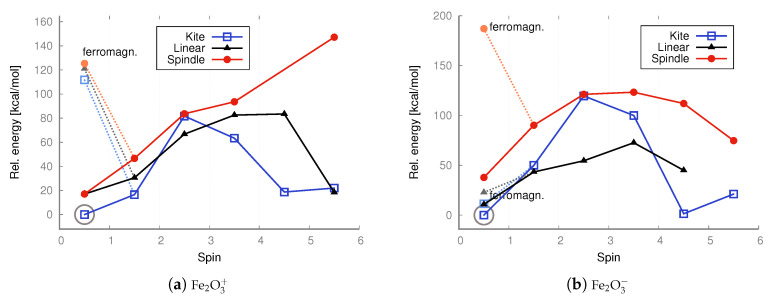
UMP2/aug−cc−pVTZ relative energies [kcal/mol] calculated with respect to the lowest-energy anti-ferromagnetic doublet (S = 1/2) state of the kite iron trioxide. Ferromagnetic (ferromagn.) doublet states are marked in lighter color. The lowest energy is marked with a circle.

**Figure 10:**
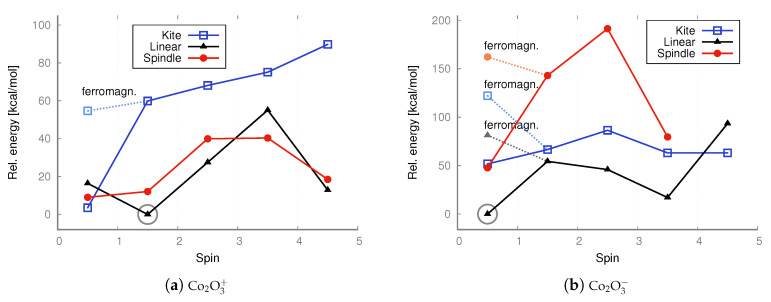
UMP2/aug−cc−pVTZ relative energies [kcal/mol] calculated with respect to the lowest-energy anti-ferromagnetic quartet (S = 3/2; cation) and anti-ferromagnetic doublet (S = 1/2; anion) states of the linear cobalt trioxide. Ferromagnetic doublet states are marked in lighter color. The lowest energy is marked with a circle.

The linear (straight or bent) geometries of Fe_2_O_3_ and Co_2_O_3_ have not previously been proposed; some authors have even claimed only one possible state exists (structure and spin) for ferric oxide [35,36,37,38,45]. However, the situation is likely more intricate, and no accurate, comprehensive depiction of the relationships between geometry, spin, and charge currently exists. Previous attempts were often based on simplistic assumptions, constructing only one type of structure, imposing limited spins, and unnecessarily applying symmetry restrictions, thus limiting hypothetical configurations. Clearly, the possible combinations of geometries, spins, and charges are numerous, and their complexity increases with particle size (Me_2_O_3_)_*n*_, where Me represents a metal atom and *n* an integer. It is crucial to identify the lowest-energy state (geometry and spin) to accurately determine physical properties and to study chemical reactions. And it is also important to compare antiferromagnetic and ferromagnetic states when so many spin combinations are possible in transition metal oxides. Thus, we considered more plausible spins for all configurations of neutral iron and cobalt trioxides and computed their energies (Figure 5 and Figure 6). At both the DFT level with the B3LYP functional and the MP2 method, we also considered antiferromagnetic electronic configurations for all iron(III) oxide and cobalt(III) oxide isomers, including their ionic variants (Figure 7, Figure 8, Figure 9 and Figure 10). We also maintained antiferromagnetic configurations following redox reactions. The optimized ionic geometries are summarized in Figure 2 and Figure 3.

As it appears from the DFT computations that the lowest-energy neutral iron trioxide is a kite-shaped nonet, ferromagnetic structure. This result differs from that obtained by Sierka et al. [35,36], who claimed that the lowest-energy iron trioxide should be a kite structure with a total spin of zero and an antiferromagnetic electronic configuration. The singlet antiferromagnetic state of kite Fe_2_O_3_ is around 10 kcal/mol higher in energy than the nonet. Taking a closer look at the energy diagrams (Figure 5 and Figure 6), it is clear that the isomers are high-open-shell singlets (antiferromagnetic) or high-spin ferromagnetic. The non-magnetic singlet state is consistently much higher in energy than the lowest-energy spin state; frequently, singlet non-magnetic states are the highest in energy. Additionally, the linear isomer in the antiferromagnetic configuration has energy similar to that of the antiferromagnetic kite. As observed (see Figure 5), the highest-spin state of iron trioxide can be a linear straight isomer or a kite structure, because their energies are almost equal (see Figure 6). Notably, linear structures had not been considered previously; our computations show that this isomer is in a very stable high-spin state, and its antiferromagnetic configuration is also very stable. When the B3LYP/aug−cc−pVTZ method was used, its energy was only approximately 10 kcal/mol higher than that of the nonet kite structure. The MP2 computations, however, indicate that the linear straight isomer is an antiferromagnetic singlet. The spindle-like structure, which appears to be the most compact of all the smallest iron trioxides (having the largest number of bonds between iron and oxygen atoms), in general, is always the highest in energy compared to the other isomers, with both antiferromagnetic and ferromagnetic electron configurations. Importantly, optimized structures could be obtained at each level of theory for every imposed spin. It is worth noting that the spindle iron trioxide was also observed in the optimized structures at the interface of α– and ϵ–Fe_2_O_3_ in the paper by Ahamed et al. [46]. Even though the energy of this type of structure at the B3LYP level of theory is relatively higher than that of the kite-like or linear straight structure, one should not exclude it from the pool of possible iron trioxide isomers; in fact, the antiferromagnetic singlet is a relatively low-energy state. The charts of energies as a function of spin show that all isomers prefer higher spins or antiferromagnetic singlet states. In summary, cobalt and iron oxides tend to favor antiferromagnetic singlets or, in contrast, high and very high spin states. Formally, a spin of 4 or 5 corresponds to, respectively, 8 or 10 singly occupied molecular orbitals. This suggests that larger stoichiometric units of iron and cobalt trioxides are likely to be very high-spin states or antiferromagnetic singlets, regardless of their spatial configurations. In general, this means that metal oxide systems have a very large number of singly occupied orbitals, with either a high or low spin (antiferromagnetic configuration). The definite configuration of a metal trioxide, regardless of size, is rigorously related to the mechanism of compound synthesis. As mentioned previously, many such methods involve wet synthesis, starting from aqueous solutions of metal salts and requiring relatively high-temperature treatment. The mechanisms of synthesizing oxides from salts are olation and oxolation, as proposed earlier based on experimental results [11,12]. Due to the lack of precise information on the configuration of products, intermediates, byproducts, and their spin configurations during olation or oxolation, a comprehensive investigation of the entire reaction pathway (from salts to oxides), including the effects of solvent and pH, is essential. One should also consider possible spin-flipping transitions. Therefore, a thorough analysis of all conceivable spatial and spin configurations and their respective ionic forms is fundamental and may enable convincing interpolation of properties, starting from the smallest possible configurations.

In the case of cobalt oxide, both DFT and MP2 calculations lead to the same conclusion: the lowest-energy isomer is the antiferromagnetic linear singlet. However, the ferromagnetic nonet (linear isomer) has almost identical energy. Also in the case of cobalt oxide, the spindle isomer, i.e., the one with the largest number of bonds (exclusively oxide bridges—no terminal oxygen atoms), is the highest-energy one.

In the case of anions and cations of Fe_2_O_3_ and Co_2_O_3_, the situation is only slightly different; see the charts in Figure 7 and Figure 9.

As revealed by the DFT computations, the lowest-energy anions and cations of iron trioxide and cobalt trioxide are the kite-shaped isomer with a spin of 1/2 (doublet state), and as indicated by the MP2 computations, the lowest-energy anionic and cationic state corresponds also to the doublet kite-shaped isomer. Antiferromagnetic configurations were preserved during redox reactions. After reduction by one electron (electron donation), the corresponding antiferromagnetic singlet states converted into doublet states with a partial antiferromagnetic electronic configuration. However, the ferromagnetic dectet (S = 9/) kite has only a few kcal/mol higher energy. However, one-electron oxidation does not lead to either a change in geometry type nor a change in electronic configuration, and the reaction product is a ferromagnetic dectet. For the spindle isomer, its energies are generally the highest among all other cationic and anionic isomers. Moreover, since the lowest-energy neutral iron trioxide is the kite-like isomer according to the DFT with the B3LYP functional computations, one must also assume that after one-electron reduction, none of the Fe–O bonds break, while the electronic configuration becomes more antiferromagnetic (it changes from the ferromagnetic nonet to antiferromagnetic doublet). It must be emphasized that for ions, all spin states were accessible for all isomers, as was the case for the neutral species. In other words, all isomers tolerate reduction or oxidation (although we have not conducted detailed studies of the reaction pathways). Moreover, as shown by both the MP2 and DFT computations, the linear isomer is very stable and, in general, only a few kcal/mol higher in energy than the corresponding spin states of the kite-shaped isomer of Fe_2_O_3_. It can also be seen that some Fe_2_$O_{3}^{+}$ isomers favor higher spin states more than the respective Fe_2_$O_{3}^{-}$ isomers. For anions and cations of cobalt oxides, the situation is similar (see the charts in Figure 7 and Figure 9). Here as well, the cations prefer larger spins, while the energy differences are not as significant as in the case of neutral iron trioxides. For anions of Co_2_O_3_, the most stable is the linear antiferromagnetic doublet (MP2 level of theory) or antiferromagnetic kite (DFT level of theory); however, energies of both linear and kite isomers are almost the same.

In addition, when a one-electron reduction or oxidation process occurs for neutral cobalt trioxide, the geometry remains linear, as found at both the DFT and MP2 levels of theory. The geometry of the linear isomer of Co_2_O_3_ becomes bent after both oxidation and reduction (see Figure S2).

**Figure 11:**
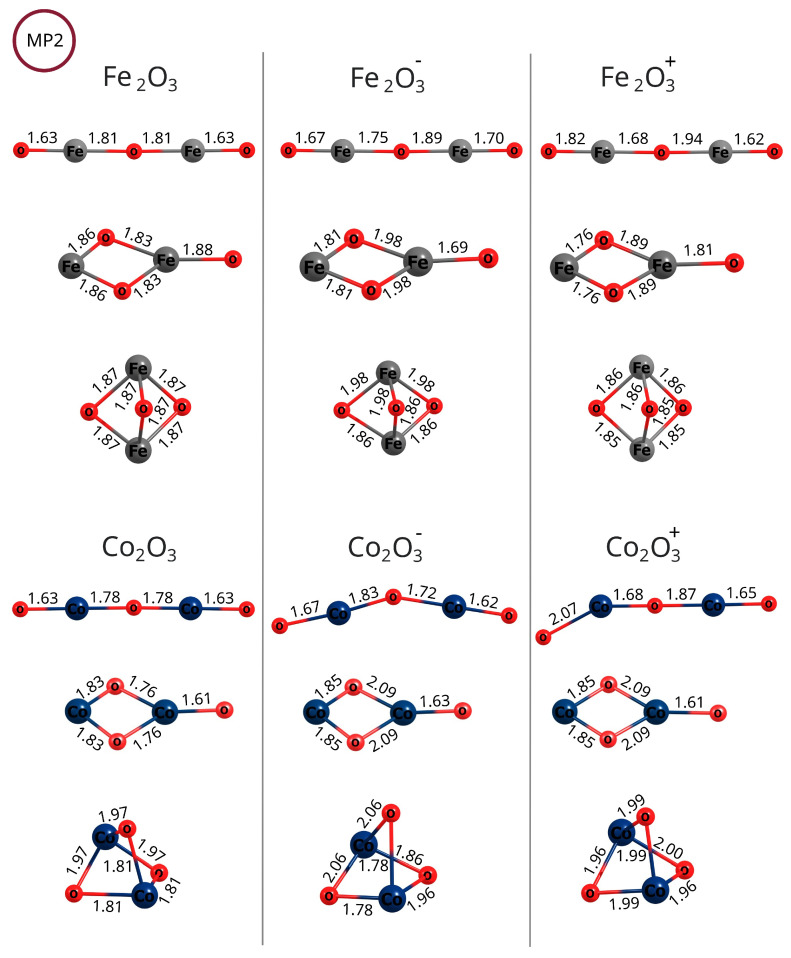
Bond lengths (Å) of the lowest-energy configurations of iron(III) and cobalt(III) oxides found at the UMP2/aug−cc−pVTZ level of theory. Antiferromagnetic states were not taken into account at this level of theory.

Comparing the geometries of the iron oxide isomers—see Figure 4 and Figure 11—it is clear that, in general, calculations at the B3LYP level of theory yield a bent uncharged and cationic linear isomer, while MP2 optimization leads to straightened linear systems. At both levels of theory, the bonds to the terminal oxygen atoms are the shortest. The kite and spindle structures of iron oxide for both neutral and ionic systems are similar, but the bonds in the anions are slightly elongated, while in the cations, they are shortened.

Similarly to the case of linear configurations, in the kite structures, the terminal oxygen atoms connect to the iron atom by the shortest bond compared to other oxygen–iron bonds. For cobalt oxide isomers, bond lengths are more uniform compared to the corresponding iron oxide isomers; that is, the bonds between the terminal oxygen atoms and iron atoms, as well as the remaining bonds, have very similar lengths. In contrast to iron oxide isomers, cobalt oxides maintain similar bond lengths after reduction or oxidation; in cations, the bonds are not significantly shortened, and in anions, the bonds are not significantly lengthened.

The calculated natural frequencies (see Tables S9 and S10 in the Supplementary Material) for all optimal isomers of iron and cobalt oxides indicate that these states are indeed local minima. Antiferromagnetic states were obtained after the full process of gradient minimization, and we checked the stability of the wave function (see the protocol for the antiferromagnetic state, provided in the Supplementary Materials).

Both calculation methods, i.e., MP2 and DFT, with an extensive basis set including a large number of polarizations and diffuse functions, lead to clearly stable isomers, also after reduction and oxidation. For each of the isomers of both cobalt(III) oxide and iron(III) oxide, there are stoichiometrically two metal atoms. Each single-electron reduction or oxidation does not cause the disintegration of the molecule, and the calculated electron affinities and ionization potentials indicate a high stability of these systems toward redox processes, similar to iron(III) and cobalt(III) salts and complexes (and other systems) used as redox couples in electrochemical cells (see Table 2, as well as Table 3).

Of course, the oxygen atom is significantly more electronegative than the iron and cobalt atoms, as described in the Methods section. Consequently, the electron density is significantly shifted toward the oxygen atoms. This is displayed by positive effective partial charges on the metal atoms and negative charges on the oxygen atoms; see Figure 2 and Figure 3. In the case of linear and spindle structures, the charges on two metal atoms are almost identical, while in the case of kite-like molecules, the charge on a metal atom bonding to three oxygen atoms is significantly lower than on a metal atom bonding to two oxygen atoms.

After single-electron oxidation (in the case of cations), the charges on the metal atoms increase slightly, while after reduction (in the case of anions), the partial charges of the metal atoms decrease slightly. This means that, after both reduction and oxidation, it is primarily the oxygen atoms that take on the ”weight” of the molecule’s new charge, while the metal atoms more readily transfer these new charges to the oxygen atoms. Of course, Hershfeld charges are significantly lower than Mulliken charges due to the more realistic charge division based on electron density rather than overlap of atomic orbitals.

#### 2.2.2. UMP2/aug−cc−pVTZ Results

The molecular structures of the UMP2/aug−cc−pVTZ–optimized local minima of the lowest-energy states are shown in Figures S10 and S11. The geometric parameters are presented in more detail in Figure 11.

At the MP2 level, we also considered both ferromagnetic and antiferromagnetic configurations. In the latter case, the starting wave function was the optimized antiferromagnetic state for the lowest spin (singlet for neutral systems and doublet for ions).

In the case of the neutral Fe_2_O_3_ system, the lowest energy structure is the antiferromagnetic linear isomer (see the energy charts in Figure 6), contrary to what was predicted by DFT calculations with the B3LYP functional, in which case the lowest energy isomer was the ferromagnetic kite with a spin of 4 (although in the case of the B3LYP calculations, the antiferromagnetic linear and kite isomers had energies similar to the ferromagnetic kite). In the case of the neutral Co_2_O_3_ system, the lowest energy structure is the antiferromagnetic linear isomer, and the same result was obtained from DFT calculations.

As revealed by the MP2 computations, the lowest-energy anionic state of iron oxide is an antiferromagnetic doublet. At the MP2 level of theory, ferromagnetic configurations were preserved during redox reactions, but the linear isomer was transformed into the kite isomer in such a process. All spin states were accessible for all isomers, as was the case for the neutral species. Hence, all isomers withstand reduction or oxidation.

For the cobalt oxides, almost all spin states were also obtained, and the same can be said about cations and anions. Moreover, as shown by both the MP2 and DFT computations, the linear isomer is very stable. In the case of the kite and spindle isomers, their energies are about 20 kcal/mol higher than the energy of the lowest-energy linear isomer (antiferromagnetic singlet). In this case, the ferromagnetic high-spin configurations are also higher in energy than the antiferromagnetic singlet configuration.

In the case of cations, the spindle configuration, as in the case of iron oxide isomers, is the least preferred because its energies are consistently the highest. In the case of cations, the higher the spin, the significantly higher the spindle energy, while both the high-spin linear and kite isomers, as well as the low-spin antiferromagnetic Isomers generally have lower energy (the system clearly prefers the high-open-shell configuration with low or high spin); see the charts in Figure 7 and Figure 9.

Additionally, when a one-electron reduction or oxidation process occurs for neutral cobalt trioxide, the geometry remains linear, as found at both the DFT and MP2 levels of theory. In the case of iron oxide, both the oxidation and reduction processes led to a change in the geometry of the system (from linear to kite). The geometry of the linear isomer of Co_2_O_3_ becomes bent after both oxidation and reduction (see Figure S2).

Comparing the geometry of the isomers of iron oxides—see Figure 4 and Figure 11—it can be seen that, in general, the calculations at the B3LYP level of theory lead to a bent uncharged and cationic linear isomer, while the MP2 optimization leads to straightened linear systems. Comparing the geometry of the lowest-energy antiferromagnetic states obtained at the MP2 level of theory, it is clear that the linear isomers of iron oxide are straightened, also after reduction and oxidation, while the linear isomers of cobalt oxide are bent. Also visible are changes in geometry, consisting, in general, of the shortening of bonds in the case of cations (relative to the systems before oxidation), while anions have relatively similar parameters compared to the corresponding systems before reduction.

### 2.3. Electrochemical Properties

In the next step, we calculated the redox properties of the studied iron and cobalt oxides, utilizing both the DFT and MP2 methods (see Table 2 and Table 3). We calculated their ionization potentials and electron affinities, both vertical and adiabatic ones. Finally, we calculated standard reduction potentials for the case of a water solvent. As mentioned before, the iron and cobalt oxides were treated as molecules, not as bulk materials. We took into account only the lowest-energy states of the neutral metal oxides, which were found in the first step (see Figure 5 and Figure 6).

The ionization potential corresponds to the oxidation reaction, while the electron affinity corresponds to the reduction, and both were calculated in a vacuum, i.e., no solvent was involved, whether more or less polar. As shown by the DFT computations (see Table 2), the vertical electron affinity (vEA) of iron(III) oxides varies from 1.21 eV for the most compact spindle isomer to 2.69 eV for a straight linear molecule, while the adiabatic EAs are generally close to each other for each of the isomers and vary from 2.36 eV to 2.77 eV, respectively. Clearly, the more compact the molecule, the smaller its electron affinity. For the MP2 computations, the vEAs range from 2.18 eV for the spindle isomer to 2.92 eV for the kite-like configuration. Similarly, the electron affinity of a neutral spindle Fe_2_O_3_ structure was observed by Hongbin Wu et al. [49] in the photoelectron spectroscopic study of monoiron oxides (Fe$O_{y}^{-}$) and diiron oxides (Fe$O_{y}^{-}$), where (*y* = 1–4) and (*y* = 1–5), respectively. The values of the adiabatic electron affinity (aEA) were determined from the binding energy of the vibrational feature at *v* = 0, when the vibrational structures were resolved for the ground state, and the reported value for the spindle at the ground state was 3.06 eV. Simultaneously, Reilly et al. reported calculated values of vertical EAs to be 2.39 eV and 2.86 eV for the singlet and triplet spin states, respectively [48]. They employed the DZVP basis set and performed calculations using the generalized gradient approximation (GGA) method. Importantly, according to their results, the ground state of Fe_2_$O_{3}^{-}$ is a doublet kite-like configuration, even though no other isomers were considered. In the case of Co_2_O_3_, the structural reactivity of cobalt oxide clusters with CO was studied by Johnson et al. [50] using DFT calculations with the generalized gradient approximation functional and DZVP basis sets. The calculated electron affinity and ionization potential for neutral Co_2_O_3_ were 1.76 eV and 7.9 eV at the antiferromagnetic configuration, respectively. However, at the quintet spin state, the calculated electron affinity was 2.75 eV and the ionization potential was 9.05 eV. Another DFT study on the Fe_2_O_*n*_ series was carried out by Gutsev et al. [47], who applied the BPW91 exchange-correlation functional and the 6-311+G′basis set of triple-*ζ* quality. At this level of theory, the lowest-energy state was identified as an antiferromagnetic singlet for 2<n<5, and the calculated adiabatic electron affinity and adiabatic ionization potential for the kite Fe_2_O_3_ were about 2.5 eV and 8.5 eV, respectively (we found it using the DFT method with the B3LYP functional in which the lowest energy isomer of iron oxide is the ferromagnetic kite with spin S = 4, and for it, the adiabatic ionization potential is 2.77 eV and the adiabatic electron affinity is 9.03 eV). Electron affinities for bulk crystal heterostructures of ϵ-Fe_2_O_3_ and α-Fe_2_O_3_ were calculated using the PBE0/pseudopotential approach with plane waves, yielding values of 4.74 eV and 3.31 eV, respectively [46]. The first ionization potentials (adiabatic), as shown in Table 2, range from about 8 eV to 9 eV for iron oxides and from about 9 eV to nearly 10 eV for cobalt oxides, as determined by the B3LYP computations. At the MP2 level of theory, the IPs and EAs are a bit higher, with the largest values for the linear isomer and the smallest for the spindle configuration. For cobalt trioxides, both the IPs and the EAs are generally higher by 1–2 eV. The ionization potential is related to the one-electron oxidation process. Accordingly, it is about three times larger than the computed electron affinities at the same level of theory, while the electron affinity corresponds to the molecule’s ability to accept an electron. Consequently, both iron(III) and cobalt(III) oxides are about three times more likely to undergo reduction than oxidation.

Although the iron oxides considered here are the smallest possible representatives of iron and cobalt oxide materials, such as nanoparticles, it has been demonstrated that they can undergo one-electron reduction. Moreover, the experimental standard reduction potential of the half-reaction ${\mathrm{Fe}}_{\mathrm{aq}}^{3+}$ + e^−^ → ${\mathrm{Fe}}_{\mathrm{aq}}^{2+}$ is +0.77 V relative to the SHE. However, under standard basic conditions, the redox potential is −0.54 V (relative to SHE). For the reaction ${\mathrm{Co}}_{\mathrm{aq}}^{3+}$ + e^−^→ ${\mathrm{Co}}_{\mathrm{aq}}^{2+}$, the standard potential is +1.82 V relative to the SHE, while under standard basic conditions, the redox potential is +0.17 V. Therefore, it is evident that aqueous cobalt(III) salts are significantly stronger oxidizers than the corresponding iron(III) salts. Iron trioxides exhibit standard reduction potentials spanning from −0.37 V to −0.72 V (relative to the SHE), depending on the geometry of the oxide, as found at the DFT level of theory (see Table 3). The largest value was calculated for the kite-like structure and the smallest for the linear isomer. From the MP2 computations, the $E_{SHE}^{\circ \;(0/-1)}$ values are similar and vary from −0.94 for the linear isomer to −0.75 for the spindle-like isomer. At the MP2 level of theory, the largest value was calculated for the spindle-like structure and the smallest for the linear isomer. For cobalt trioxides, the standard reduction potentials span from −0.63 V to −0.18 V, as determined by the DFT calculations. After the MP2 computations, the standard reduction potentials are more diverse and range from −2.16 V to +0.75 V, Thus, despite the fact that (Me_2_O_3_)_1_ molecules are the smallest possible units, it is justified to claim that iron and cobalt trioxides demonstrate redox behavior such that they preferentially undergo reduction. Cobalt oxide is more susceptible to reduction than iron oxide. The same conclusion applies to the aqueous salts of cobalt(III) and iron(III). It is clear that kite Fe_2_O_3_ and linear Co_2_O_3_ are the most easily reduced by one electron, in contrast to the other respective configurations, as revealed from the DFT investigations. By analogy, the kite configuration of iron oxide and the linear molecule of cobalt oxide are the least susceptible to oxidation, compared to the other configurations, because they have the largest ionization potentials. Interestingly, the linear configuration of iron(III) oxide was not even mentioned by the authors investigating possible configurations of iron trioxides, whether smaller or larger [35,36,37,38,45]. The investigations of larger systems partially support our results. The standard potential of the iodide/triiodide redox couple is 0.35 V (versus the SHE) [51], and this redox couple is frequently used, for instance, in dye-sensitized solar cells. Fe_2_O_3_ small hematite crystals possess photocatalytic activity for the oxidation of sulfite (S(IV)), as shown by Faust et al. [52]. Kormann et al. [53] examined the usability of Fe_2_O_3_ (3–20 nm in size) as a photocatalyst. They also compared the photocatalytic activity of hematite to that of colloids and suspensions of ZnO and TiO_2_ [54]. Furthermore, Yan et al. demonstrated that the pseudocapacitive behavior of Fe_2_O_3_ can occur in 1-ethyl-3-methylimidazolium tetrafluoroborate (EMIMBF_4_) ionic liquid, and it is closely related to the chemical state variation between Fe^3+^ and Fe^2+^ on the surface of an Fe_2_O_3_ electrode during charging/discharging processes [22]. Interestingly, the presence of an antiferromagnetic configuration or a high-spin ferromagnetic configuration does not significantly affect the calculated redox potentials when the same isomer is considered; the calculated redox potential values are quite similar.

## 3. Methods

### 3.1. Iron(III) and Cobalt(III) Oxides’ Spatial and Electron Configuration

In general, the bonds between iron and oxygen atoms, as well as between cobalt and oxygen atoms, are of the covalent type. This is because the difference between the electronegativities of iron and oxygen, as well as those of cobalt and oxygen, does not exceed 1.7 [55]. In fact, for transition metal oxides, the nature of the metal–oxygen bond is widely considered to be covalent, nearly ionic, and significantly polarized towards oxygen.

For iron and cobalt oxides, the oxo ion, i.e., O^2−^, does not influence the total spin of the oxide if one could deduce the total spin number simply by adding the contributions from each ion independently. However, in molecules containing covalent bonds, molecular orbitals—not atomic ones—are present. Furthermore, for oxygen–oxygen bonds (as in the dioxygen molecule, which could be considered an oxygen oxide) and oxygen–metal bonds, we observe not one but two π bonding and two π antibonding orbitals. Moreover, since transition metals such as iron and cobalt can have many 3D sublevels that are partially occupied, it is advisable to assume that some of the states may remain nonbonding. As a result, for metal oxides, the only way to determine the total spin of a given metal oxide is to impose all hypothetically possible spins and investigate their energetics. Moreover, among transition metal compounds, including oxides, there is a relatively common antiferromagnetic configuration in which α and β spins are arranged non-equivalently such that, on individual metal atoms, one of the spins is dominant, while the neighboring metal atom has the opposite spin. The resulting spin is then zero, and the system is non-magnetic. Such a configuration is technically possible using multi-configuration methods (open-shell singlet configuration of the singlet state or as the “core” of a high-spin system), and also using the DFT method in the Gaussian program [56] by defining spin orbitals within the DODS approximation (unrestricted HF method). On this basis, we imposed the following spins: {0,1,2,3,4,5} on the neutral iron trioxides and {0,1,2,3,4} for neutral cobalt trioxides. For the case of all spins, we imposed both (independently) ferromagnetic (or for spin S = 0 nonmagnetic) and antiferromagnetic configurations. In the latter case, we first performed calculations for the highest-spin system (S=5 for iron oxides and S=4 for cobalt oxides) and then used the resulting wave function as the starting point for calculations with the low-spin antiferromagnetic configuration. After ensuring that the electron configuration indeed corresponded to an antiferromagnetic singlet state, we then used this final configuration for all non-singlet states (for each spin), thereby forcing part of the initial electron configuration to be antiferromagnetic. These states then underwent a geometry optimization process. For systems, after one-electron reduction or oxidation, we did the same, but in the case of reduction or oxidation of a low-spin antiferromagnetic system, we assumed that this configuration was preserved during the redox process. In such cases, we also imposed an antiferromagnetic configuration for ionic states. Then, the initial orbitals came from calculations for high-spin metal oxide ions: S=11/2 for Fe_2_ $O_{3}^{+}$ and S=9/2 for Co_2_$O_{3}^{+}$. A complete example of the entire calculation protocol for the antiferromagnetic state is provided in the supplementary materials, along with the definition of the input variables and output file data.

Furthermore, we designed three different types of geometries for the smallest possible iron and cobalt trioxides, as shown in the results section. The constraints were the stoichiometry of the oxides, as well as the requirement that the molecules be of an alternating type. We also did not impose symmetry in order to obtain the lowest-energy structures, despite the fact that previous studies on selected isomers of iron(III) oxides almost always used symmetry constraints for both the geometry and the wave function/electron density. Regarding the quantum methods, we chose not to use Bloch plane-wave basis functions, since the molecular orbitals of oxides should be quite localized, because the metal and oxygen atoms have very different electronegativities [57]. Instead, we used a correlation-consistent polarized basis set, aug−cc−pVTZ, a basis set that systematically converges toward the complete basis set, which is rich in polarization functions (separate sets for the description of valence 3d4s electron correlation) and augmented by diffuse functions in each orbital angular momentum necessary for ion computations [58,59]. Some initial geometries were also generated using the Effective Core Potential (ECP) plus DZ basis set, LANL2DZ [56].

The equilibrium geometries and their properties were identified using the post Hartree–Fock Møller–Plesset second-order (MP2) perturbation method, as well as the density functional method, along with two exchange-correlation functionals: B3LYP and BHHLYP [60,61,62,63,64]. B3LYP is a hybrid combining five functionals: Becke, Slater, HF exchange (B3), LYP and VWN5 correlation. BHHLYP (Becke’s half-and-half (BH&H)) is a combination of three functionals: 0.5 HF, 0.5 Becke88, and LYP. To construct the orbitals, we used both restricted and unrestricted approaches to test whether low-spin and high-spin states would be characterized by spin contamination and to qualitatively assess both approaches. The advantage of DFT methods over post-Hartree–Fock methods is the lower computational cost for dynamic correlation energy. The B3LYP functional has become the standard hybrid density functional for studying gas-phase chemistry, as it offers a good compromise between computational cost, coverage, and accuracy [65]. Both the DFT with the usage of the B3LYP functional and the DFT with the usage of the BHHLYP functional methods were recently validated for computing the redox properties of polyoxometalates [43]. On the other hand, we also used the MP2 method with the aug−cc−pVTZ basis set to evaluate the performance of both methods and to achieve a better description of the electronic structure. In both methods, we took into account ferro- and antiferromagnetic configurations, as described above, while in the case of MP2 calculations, the starting antiferromagnetic configuration was obtained at the DFT/B3LYP level of theory, in the case of neutral systems or singlets and in the case of ions for doublets, in a similar way as in the case of DFT/B3LYP calculations. Moreover, because the presence of transition metals, particularly iron, frequently caused convergence issues with the SCF algorithm, we often used the Pulay (DIIS) method [66], followed by approximate second-order quasi-Newton SCF orbital optimization [67,68] and sometimes supplemented with level shifting [69].

A population analysis of partial charges was also performed, namely Mulliken and Hirshfeld. Mulliken analysis assigns charges based on the overlap of atomic orbitals and is, thus, basis-set-dependent. Hirshfeld analysis, on the other hand, uses a partitioning scheme based on the molecular electron density, resulting in charges that are generally smaller, less basis-set-dependent, and more physically reasonable [70,71].

To confirm that the identified optimal isomer geometries are indeed local minima, we calculated the Hessian for each case to determine if any natural frequencies corresponded to negative eigenvalues after diagonalizing the Hessian.

### 3.2. Derivation of Electrochemical Quantities

**Figure 12:**
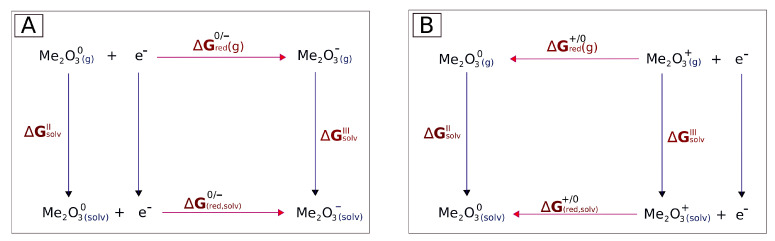
Born–Haber cycle adapted for 2 kinds of one-electron reduction reactions studied in this work: (**A**) reduction of iron(III) oxides and cobalt(III) oxides and (**B**) their cationic states. Gibbs free energies (ΔG) were computed after the optimization of the structures in a vacuum (g) and after the involvement of a solvent (solv) within the Polarization Continuum Model, as described in the Methods section. $\Delta G_{\mathrm{solv}}^{II}$ denotes the solvation free energy of a neutral system. $\Delta G_{\mathrm{solv}}^{III}$ denotes the solvation free energy of the cation or the anion (these two energies are different). $\Delta G_{\mathrm{red}}^{+/0}$ and $\Delta G_{\mathrm{red}}^{0/-}$ denote the free energies of the reduction reaction of a cation to a neutral molecule and of a neutral molecule to an anion, respectively.

Li and co-workers developed a density functional theory protocol for calculating standard redox potentials of copper complexes, and they calculated the potentials in terms of the Gibbs free energy change of the redox reaction [39]. A similar approach was used for the calculation of reduction potentials for ferrocene, actinide, and other metal complexes [39,72,73,74,75,76]. Essentially, this method involves the Born–Haber cycle adapted for redox reactions, which in turn is an application of Hess’s Law. In this work, we adapted this protocol to calculate the standard reduction potentials for two types of reactions: (1) Me_2_O_3_ + e^−^ → Me_2_$O_{3}^{-}$ and (2) Me_2_$O_{3}^{+}$ + e^−^ → Me_2_O_3_, as depicted in Figure 12.

Each reactant and product in the spin state exhibiting the lowest energy (see the Results section), i.e., the neutral molecules, as well as their cations and anions, were first optimized in a vacuum. If the lowest-energy state was the state with the antiferromagnetic configuration, then this state was taken into account. In the next steps, the effects of the surrounding water (the solvent) were approximated by employing the single-step conductor-like polarized continuum solvation model (C-PCM) within a self-consistent reaction field treatment, as implemented in the GAUSSIAN and GAMESS software packages [56,77,78]. The Gibbs free energy changes (ΔG) for the reactions in the gas phase, as well as the Gibbs free solvation energies, were calculated at a temperature of 298.15 K and a pressure of 1013 hPa (1 atm). For the Gibbs free solvation energy, we used the relative permittivity of water, $\epsilon_{r}$. Subsequently, the absolute standard redox (reduction) potential was calculated as

(1) $$E^{0}=-\frac{\Delta G_{\left(\mathrm{solv}\right)}}{nF}$$

where *n* denotes the number of electrons (here, n=1) and *F* is the Faraday constant. The standard reduction potential measures the tendency of a chemical substance to lose or gain electrons. This measurement is performed under standard thermodynamic conditions, i.e., pH 0.0, 1 M [H^+^], 25 °C, and 1 atmosphere pressure. Different compounds have distinct potentials for donating or accepting electrons. The standard reference reaction for such comparisons is the Standard Hydrogen Electrode (SHE), in which the hydrogen molecule’s dissociation process, H_2_ ⇌ 2H^+^ + 2e^−^, is assigned an absolute standard potential of +4.44 V. Thus, the release of electrons by hydrogen is used as a standard against which the tendency of other compounds to release or accept electrons is measured ($E_{\mathrm{SHE}}^{0}$). Therefore, we also present the values of

(2) $$E_{\mathrm{SHE}}^{0\;\left(\mathrm{reaction}\right)}=E^{0\;\left(\mathrm{reaction}\right)}-E_{\mathrm{SHE}}^{0}$$

We additionally calculated the electron affinities and ionization potentials, both vertical and adiabatic forms.

## 4. Conclusions

Nanoparticles, for instance, those composed of transition metal oxides, are essentially large molecules. In some segments of their structure, they can be periodic (“crystal”), but not necessarily. A theoretical description of these structures is difficult, because the possible number of stoichiometric spatial configurations is quite large and only a small subset may exhibit symmetry. Thus, any symmetry imposition would automatically restrict the analysis and lead to loss of information about the real, lowest-energy structures. This was evident in our studies. We showed that a deeper analysis of the stoichiometric geometries and spins of iron(III) and cobalt(III) oxides lead to new conclusions. That is, the lowest-energy iron trioxide is a ferromagnetic nonet (S = 4) kite isomer, as revealed by the B3LYP/aug−cc−pVTZ computations. However, the antiferromagnetic singlet is also a very low-energy configuration, only about 10 kcal/mol higher in energy than the nonet state. In general, it is clear that iron(III) oxide isomers are most stable when the spin is high or when the configuration is antiferromagnetic. In both cases, there are a large number of singly occupied molecular orbitals. Hence, these results are different from those obtained previously by Sierka et al. [35,36], who claimed that the lowest-energy iron trioxide should be a kite structure with the antiferromagnetic electronic configuration. We found that low-spin non-magnetic states of ferric oxide are evidently very unstable compared to high-spin or antiferromagnetic states, regardless of the quantum-chemical methods employed. We confirmed the same conclusion even using spin-restricted Kohn–Sham orbitals for ferromagnetic (only) states. For cobalt trioxide, the lowest-energy state is the antiferromagnetic linear isomer, but the straight linear nonet isomer is also a low-energy isomer, as found in the DFT computations. Neither a spatial nor a spin configuration should be deliberately neglected, because one cannot exclude spin or spatial transitions in reaction pathways, synthesis, or other processes. On the other hand, if the antiferromagnetic configuration is excluded from consideration, the energy differences between high and low spin states remain relatively large. On this basis, one can conclude that the final particles and the intermediate oxides formed during the synthesis of cobalt(III) and iron(III) oxide nanosystems are most likely very high-spin states or antiferromagnetic. For cations and anions formed after one-electron redox reactions, the situation is basically similar; i.e., higher-spin states are preferred in all cases, or possibly the antiferromagnetic configuration. Fe_2_ $O_{3}^{+}$ isomers prefer higher spin states than Fe_2_$O_{3}^{-}$ isomers, while for cobalt oxides, the situation is the opposite.

The one-electron reduction properties of cobalt and iron trioxides are relatively similar, but the calculated ionization potentials, electron affinities, and standard reduction potentials are generally higher for cobalt oxides. Their calculated standard one-electron reduction potentials are lower than the potentials commonly reported in textbooks for aqua salts, despite the fact that the configuration influences the calculated quantities. The most vulnerable to one-electron reduction are kite Fe_2_O_3_ and Co_2_O_3_ molecules. By contrast, the linear configurations of iron oxide and cobalt oxide are the least susceptible to oxidation because they exhibit the largest ionization potentials. We believe that the involvement of larger configurations of iron and cobalt trioxides, i.e., the configurations of (Me_2_O_3_)_*n*_ molecules for larger *n*, should reveal similar redox properties for oxides of this kind, provided that comprehensive and convincing studies of all possible spin and spatial configurations are conducted in the first place. Such a process is currently challenging due to the dependence of the results on the quality of the quantum-chemistry algorithms, including accurate representation of the ionic states.

## Figures and Tables

**Table 1:** MP2/aug−cc−pVTZ and B3LYP/aug−cc−pVTZ values of vertical Electron Affinity (vEA) and adiabatic Electron Affinity (aEA) for the FeO molecule. The values are given in eV. EA^exper.^ = 1.4950 eV [40]. EA^theor.^ = 1.52 eV [41].

FeO/FeO^−^							
vEA		aEA		vEA		aEA	
UMP2	RMP2	UMP2	RMP2	UB3LYP	RB3LYP	UB3LYP	RB3LYP
1.35	1.44	1.52	1.54	0.84	0.89	1.17	1.22

**Table 2:** MP2/aug−cc−pVTZ and B3LYP/aug−cc−pVTZ values of vertical Ionization Potential (vIP), adiabatic Ionization Potential (aIP), vertical Electron Affinity (vEA), and adiabatic Electron Affinity (aEA) for Fe_2_O_3_ and Co_2_O_3_. The values are given in eV. The left-side superscript *m* in ^*m*^calc represents the values calculated at the spin state *m*. The right-side superscripts refer to the literature data.

	vIP		aIP		vEA		aEA			
	MP2	B3LYP	MP2	B3LYP		MP2	B3LYP	MP2	B3LYP	
Fe_2_O_3_					Expt. ^m^calc.					Expt. ^m^calc.
linear	11.18	9.74	10.26	9.46	^1^ 8.5 [47]	2.37	2.69	2.56	2.76	^1^ 2.39, ^3^ 2.86 [48] ^1^ 2.6, ^3^ 3.06 [49]
kite	10.25	9.31	9.30	9.03		2.92	2.12	3.24	2.77	
spindle	9.32	8.52	9.16	8.21		2.18	1.21	2.49	2.36	
Co_2_O_3_										
linear	12.42	10.19	11.11	9.89	^6^ 9.05 [50]	2.44	2.76	2.53	2.79	^6^ 2.75 [50]
kite	11.17	10.37	9.86	9.37		1.03	2.23	1.69	2.94	
spindle	9.92	9.12	9.25	8.69		2.54	1.35	2.71	2.47	

**Table 3:** UMP2/aug−cc−pVTZ and UB3LYP/aug−cc−pVTZ values of absolute standard reduction potentials (E^∘^) and standard reduction potentials calculated against the SHE ($E_{SHE}^{\circ }$) for the lowest-energy states of Fe_2_O_3_ and Co_2_O_3_ The temperature was equal to 298.15 K and the pressure was equal to 1013 hPa (1 atm). Relative water permittivity $\epsilon_{r}$ = 80. The notation (+1/0) corresponds to the reaction of reduction of the cation to the neutral molecule, and (0/−1) corresponds to reaction of reduction of a neutral molecule to an anion. The values are given in V.

	$E^{\circ \;(+1/0)}$		$E_{\mathrm{SHE}}^{\circ \;(+1/0)}$		$E^{\circ \;(0/-1)}$		$E_{\mathrm{SHE}}^{\circ \;(0/-1)}$	
	MP2	B3LYP	MP2	B3LYP	MP2	B3LYP	MP2	B3LYP
Fe_2_O_3_								
linear (ant.)	5.90	5.77	1.46	1.33	3.50	3.61	−0.94	−0.83
kite	5.42	4.54	0.98	0.10	3.66	4.07	−0.78	−0.37
spindle (ant.)	5.42	4.77	0.98	0.33	3.69	3.72	−0.75	−0.72
Co_2_O_3_								
linear (ant.)	7.26	6.57	2.82	2.13	4.43	3.81	−0.01	−0.63
kite	6.14	6.14	1.70	1.70	2.28	4.26	−2.16	−0.18
spindle	5.12	5.12	0.68	0.68	5.19	4.12	0.75	−0.32

## Data Availability

The original contributions presented in this study are included in the article/Supplementary Material. Further inquiries can be directed to the corresponding author.

## Supplementary Materials

The following supporting information can be downloaded at: https://www.mdpi.com/article/10.3390/molecules30214158/s1, Figure S1: Molecular structures of UMP2/aug−cc−pVTZ–optimized the lowest energy states of iron trioxides and the corresponding cations and anions. Total spins (S) were shown for each optimized state. No symmetry was imposed on the geometry. The structures were optimized in vacuum. Mulliken (M) and Hirshfeld (H) partial charges were shown in parentheses for the iron atom. Spin density values are shown in blue. If a given lowest-energy state among all those considered was characterized by an antiferromagnetic arrangement of spins, then this was marked in the drawing; Figure S2: Molecular structures of UMP2/aug−cc−pVTZ–optimized the lowest energy states of cobalt trioxides and the corresponding cations and anions. Total spins (S) were shown for each optimized state. No symmetry was imposed on the geometry. The structures were optimized in vacuum. Mulliken (M) and Hirshfeld (H) partial charges were shown in parentheses for the iron atom. Spin density values are shown in blue. If a given lowest-energy state among all those considered was characterized by an antiferromagnetic arrangement of spins, then this was marked in the drawing; Figure S3: RBHHLYP/aug−cc−pVTZ relative energies [kcal/mol] calculated with respect to the lowest-energy states of iron trioxide (left panel) and cobalt trioxide (right panel); Table S1: Lowest energy configurations of Fe_2_O_3_ isomers. B3LYP/aug−cc−pVTZ level of theory; Table S2: Lowest energy configurations of Co_2_O_3_ isomers. B3LYP/aug−cc−pVTZ level of theory; Table S3: Lowest energy configurations of Fe_2_O_3_ isomers. UMP2/aug−cc−pVTZ level of theory; Table S4: Lowest energy configurations of Co_2_O_3_ iosmers. UMP2/aug−cc−pVTZ level of theory.; Table S5: Spin contamination for ground state configurations of Fe_2_O_3_ at the UB3LYP/aug−cc−pVTZ level of theory; Table S6: Spin contamination for ground state configurations of Fe_2_O_3_ at the UMP2/aug−cc−pVTZ level of theory; Table S7: Spin contamination for ground state configurations of Co_2_O_3_ at the UB3LYP/aug−cc−pVTZ level of theory; Table S8: Spin contamination for ground state configurations of Co_2_O_3_ at the UMP2/aug−cc−pVTZ level of theory; Table S9: Normal vibration frequencies and IR intensities of equilibrium states of Fe_2_O_3_ found at the UB3LYP/aug−cc−pVTZ level of theory. Freqencies are given in cm^−1^ and IR intensities in Debye^2^/amu-Angstrom^2^ units; Table S10: Normal vibration frequencies and IR intensities of equilibrium states of Co_2_O_3_ found at the UB3LYP/aug−cc−pVTZ level of theory. Freqencies are given in cm^−1^ and IR intensities in Debye^2^/amu-Angstrom^2^ units; Table S11: Normal vibration frequencies and IR intensities of equilibrium states of Fe_2_O_3_ found at the UMP2/aug−cc−pVTZ level of theory. Freqencies are given in cm^−1^ and IR intensities in Debye^2^/amu-Angstrom^2^ units; Table S12: Normal vibration frequencies and IR intensities of equilibrium states of Co_2_O_3_ found at the UMP2/aug−cc−pVTZ level of theory. Freqencies are given in cm^−1^ and IR intensities in Debye^2^/amu-Angstrom^2^ units; Table S13: RMP2/aug−cc−pVTZ and RB3LYP/aug-c-pVTZ relative energies of states of FeO molecule; Table S14: RMP2/aug−cc−pVTZ and RB3LYP/aug-c-pVTZ relative energies of states of FeO^−^.
