# Supplementary material for: Isomers of Iron(III) Oxides and Cobalt(III) Oxides and Their Redox Properties: Quantum-Chemical Insights
Source: Molecules. 2025 Oct 22;30(21):4158. doi: 10.3390/molecules30214158 (PMC12608324; doi:10.3390/molecules30214158)
Supplement: Supplementary file 1 [file molecules-30-04158-s001.zip › molecules-3589432-supplementary.pdf]

Supplementary Materials: Isomers of Iron(III) Oxides and Cobalt(III) Oxides and Their Redox Properties: Quantum-Chemical Insights

Sapajan Ibragimov [1,2], Leonard Komando [3] and Maciej Bobrowski [3,*]

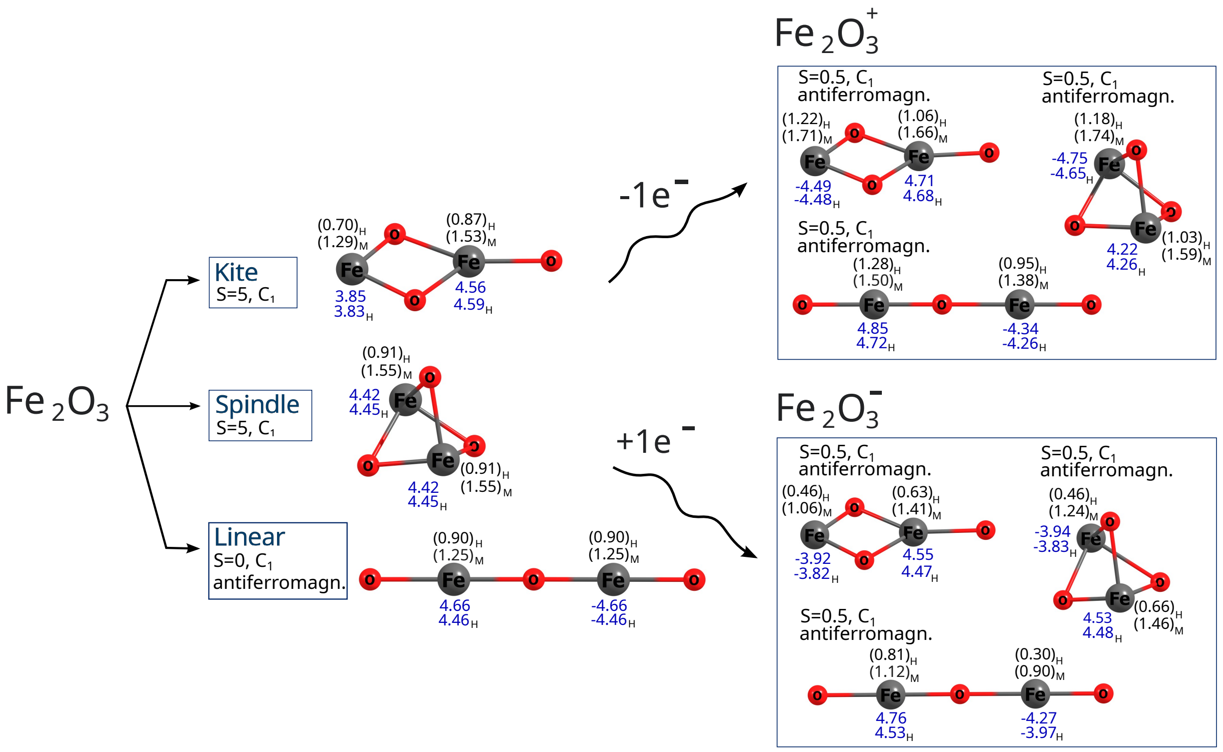

Figure S1. Molecular structures of UMP2/aug−cc−pVTZ–optimized the lowest energy states of iron trioxides and the corresponding cations and anions. Total spins (S) were shown for each optimized state. No symmetry was imposed on the geometry. The structures were optimized in vacuum. Mulliken (M) and Hirshfeld (H) partial charges were shown in parentheses for the iron atom. Spin density values are shown in blue. If a given lowest-energy state among all those considered was characterized by an antiferromagnetic arrangement of spins, then this was marked in the drawing.

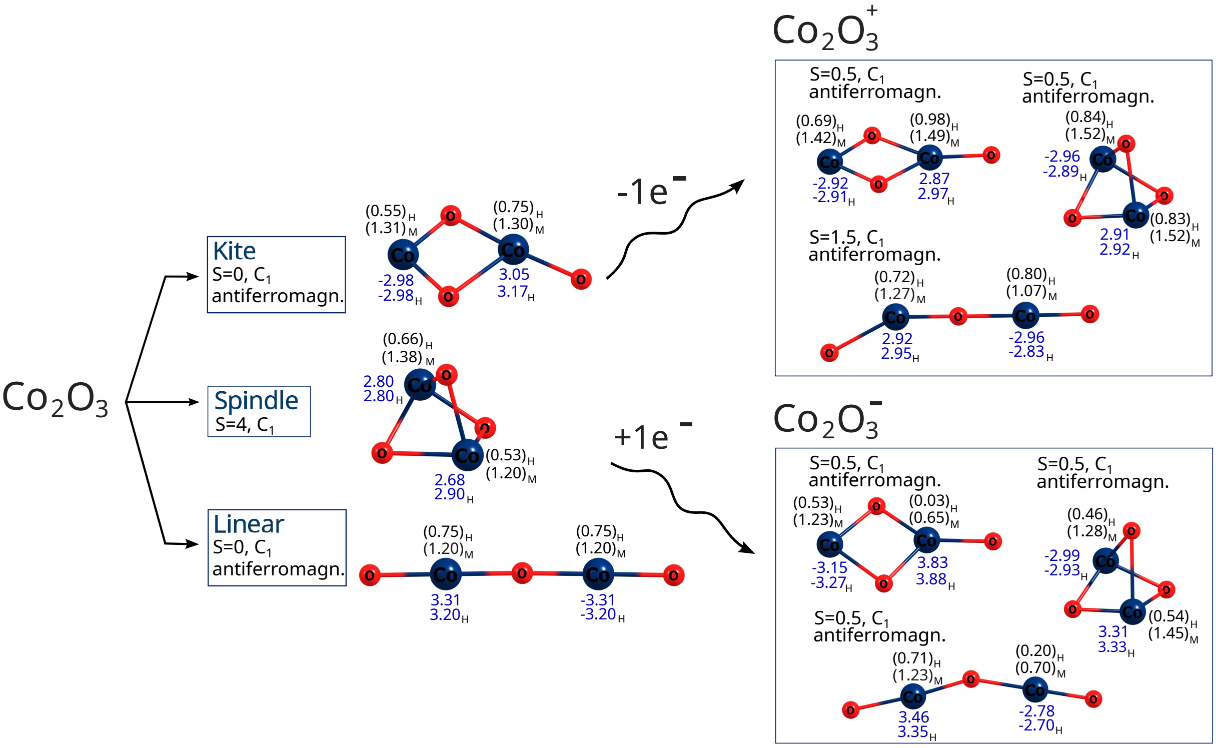

Figure S2. Molecular structures of UMP2/aug−cc−pVTZ–optimized the lowest energy states of cobalt trioxides and the corresponding cations and anions. Total spins (S) were shown for each optimized state. No symmetry was imposed on the geometry. The structures were optimized in vacuum. Mulliken (M) and Hirshfeld (H) partial charges were shown in parentheses for the iron atom. Spin density values are shown in blue. If a given lowest-energy state among all those considered was characterized by an antiferromagnetic arrangement of spins, then this was marked in the drawing.

Table S1. Lowest energy configurations of Fe_2O_3 isomers. B3LYP/aug−cc−pVTZ level of theory.

Kite	Fe_2O_3			$Fe_2O_3^-$			$Fe_2O_3^+$		
Atom Nr.	X	Y	Z	X	Y	Z	X	Y	Z
26.0	0.000000	0.000000	-0.680752	-0.000000	0.000000	-0.695184	-0.000000	0.000000	-0.744510
26.0	-0.000000	-0.000000	1.879110	0.000000	-0.000000	1.756368	0.000000	-0.000000	1.881222
8.0	0.000000	0.000000	-2.317032	-0.000000	0.000000	-2.389332	-0.000000	0.000000	-2.418062
8.0	-1.290264	-0.000000	0.559336	-1.410232	-0.000000	0.664073	-1.272818	-0.000000	0.640675
8.0	1.290264	-0.000000	0.559336	1.410232	-0.000000	0.664073	1.272818	-0.000000	0.640675
Spindle<br>Atom Nr.									
8.0	-0.000583	1.492168	0.000000	-0.001431	1.605861	-0.036569	1.451828	-0.000057	-0.000049
26.0	0.000279	-0.000165	1.112040	0.017908	0.022502	1.046810	0.000059	0.000102	1.208454
8.0	-1.292535	-0.745576	0.000000	-1.389400	-0.804442	0.044300	-0.725982	1.257275	-0.000055
26.0	0.000279	-0.000165	-1.112040	-0.018359	-0.021947	-1.045991	-0.000032	-0.000067	-1.208431
8.0	1.292559	-0.746263	0.000000	1.391283	-0.801974	-0.008551	-0.725866	-1.257270	0.000066
Linear<br>Atom Nr.									
8.0	0.000000	0.000000	3.451335	0.000000	0.000000	3.479255	0.000000	0.000000	3.446301
26.0	0.000000	0.000000	1.799494	0.000000	0.000000	1.797775	0.000000	0.000000	1.795708
8.0	0.000000	0.000000	0.000000	0.000000	0.000000	0.044921	0.000000	0.000000	-0.103891
26.0	0.000000	0.000000	-1.799494	0.000000	0.000000	-1.811601	0.000000	0.000000	-1.769418
8.0	0.000000	0.000000	-3.451335	0.000000	0.000000	-3.479243	0.000000	0.000000	-3.427852

Table S2. Lowest energy configurations of Co_2O_3 isomers. B3LYP/aug−cc−pVTZ level of theory.

Kite	Co_2O_3			$Co_2O_3^-$			$Co_2O_3^+$		
Atom Nr.	X	Y	Z	X	Y	Z	X	Y	Z
27.0	-0.000000	-0.000000	1.563739	-0.000000	0.000000	1.472748	-0.000000	-0.000000	1.540925
27.0	0.000000	0.000000	-0.955000	0.000000	-0.000000	-0.951579	0.000000	0.000000	-0.979050
8.0	0.000000	1.284169	0.254543	-0.000000	1.427555	0.428773	0.000000	1.281693	0.342986
8.0	-0.000000	-1.284169	0.254543	-0.000000	-1.427555	0.428773	-0.000000	-1.281693	0.342986
8.0	0.000000	0.000000	-2.563579	0.000000	-0.000000	-2.616491	0.000000	0.000000	-2.582301
Spindle Atom Nr.									
8.0	0.085715	-1.052561	-1.091449	0.336231	-1.358706	0.738999	0.002072	0.918600	-1.177725
27.0	1.115680	0.000045	0.000312	1.051985	0.000298	-0.138905	-1.152504	-0.052076	0.000079
8.0	0.086072	1.471502	-0.364503	-0.374273	-0.000962	-1.503404	-0.003757	-1.480049	0.000631
27.0	-1.191282	0.000387	-0.000571	-1.139808	-0.000206	0.146801	1.152527	-0.054375	0.000220
8.0	0.083371	-0.420400	1.456824	0.334442	1.359358	0.737758	0.001610	0.920721	1.176085
Linear Atom Nr.									
8.0	0.000000	0.000000	3.398110	0.000000	0.000000	3.425347	0.000000	0.000000	3.441997
27.0	0.000000	0.000000	1.765299	0.000000	0.000000	1.767655	0.000000	0.000000	1.817887
8.0	0.000000	0.000000	-0.000001	-0.000000	-0.000000	0.000000	0.000000	0.000000	0.000000
27.0	0.000000	0.000000	-1.765298	0.000000	0.000000	-1.767655	0.000000	0.000000	-1.817887
8.0	0.000000	0.000000	-3.398110	0.000000	0.000000	-3.425347	0.000000	0.000000	-3.441997

Table S3. Lowest energy configurations of Fe_2O_3 isomers. UMP2/aug−cc−pVTZ level of theory.

Kite	Fe_2O_3			$Fe_2O_3^-$			$Fe_2O_3^+$		
Atom Nr.	X	Y	Z	X	Y	Z	X	Y	Z
26.0	-0.000000	0.000000	-0.822637	0.000000	-0.000000	-0.719442	0.000000	0.000000	-0.690426
26.0	0.000000	-0.000000	1.808745	0.000000	-0.000000	1.789549	-0.000000	0.000000	1.847029
8.0	-0.000000	0.000000	-2.452774	-0.000000	-0.000000	-2.405178	-0.000000	0.000000	-2.503729
8.0	-1.353552	-0.000000	0.733332	-1.415628	0.000000	0.667534	-1.305216	-0.000000	0.673562
8.0	1.353552	-0.000000	0.733332	1.415628	0.000000	0.667535	1.305216	-0.000000	0.673562
Spindle Atom Nr.									
8.0	-0.003417	-0.001271	0.019677	0.000316	1.570044	0.042515	0.000285	1.432529	0.002258
26.0	0.018393	0.028369	1.885868	0.000250	-0.000144	1.034565	-0.000249	0.000128	1.173743
8.0	1.883998	0.021411	1.782692	-1.359541	-0.785296	0.042515	-1.240484	-0.716495	0.002256
26.0	1.305077	1.247640	0.496623	-0.000699	0.000402	-1.163248	-0.000019	-0.000008	-1.180283
8.0	0.105150	1.893777	1.771237	1.359673	-0.785008	0.043653	1.240466	-0.716156	0.002026
Linear Atom Nr.									
8.0	-0.000870	-0.000000	0.131799	-0.000729	-0.000000	0.097238	-0.001123	-0.000131	0.009960
26.0	0.003577	0.000000	1.766713	0.003354	0.000000	1.763016	0.003758	0.000244	1.833915
8.0	0.012246	0.000000	3.579943	0.010970	-0.000000	3.514092	0.011392	0.000001	3.517958
26.0	0.020036	-0.000000	5.393178	0.021517	-0.000000	5.411626	0.020961	-0.000219	5.460017
8.0	0.031115	0.000000	7.028060	0.030993	0.000000	7.113722	0.031117	0.000106	7.077844

Table S4. Lowest energy configurations of Co_2O_3 iosmers. UMP2/aug−cc−pVTZ level of theory.

Kite	Co_2O_3			$Co_2O_3^-$			$Co_2O_3^+$		
Atom Nr.	X	Y	Z	X	Y	Z	X	Y	Z
27.0	-1.059217	-0.000004	0.000697	0.000004	-0.060489	1.662613	-1.172707	-0.000061	-0.000000
27.0	1.357438	0.000012	-0.000894	-0.000004	-0.079483	-1.048446	1.297045	0.000032	-0.000000
8.0	-0.161718	1.371451	-0.000061	-0.000002	1.462333	0.322572	0.006839	1.262664	0.000000
8.0	-0.161717	-1.371451	-0.000059	-0.000002	-1.319137	0.298641	0.006804	-1.262613	0.000000
8.0	3.125215	-0.000007	0.000317	0.000004	-0.003225	-2.681133	2.962018	-0.000022	-0.000000
Spindle Atom Nr.									
8.0	1.505171	-0.001085	0.053239	1.555832	-0.051098	0.095130	0.012081	-1.344065	-0.699976
27.0	0.000368	0.002106	1.062262	0.031868	-0.056056	1.012483	-1.255259	-0.003205	0.052744
8.0	-0.752963	1.303801	0.053046	-0.795510	1.374218	-0.034662	-0.014676	0.003915	1.575597
27.0	-0.000833	-0.001042	-1.224452	-0.059079	0.106455	-1.172226	1.256606	-0.015829	0.028776
8.0	-0.751744	-1.303780	0.055903	-0.733111	-1.373519	0.099275	0.001277	1.357531	-0.684669
Linear Atom Nr.									
8.0	0.000000	-0.000000	-3.407012	0.000000	0.000000	-3.400380	-0.000000	0.000000	-3.523390
27.0	-0.000000	0.000000	-1.779611	-0.000000	-0.000000	-1.775508	0.000000	-0.000000	-1.873260
8.0	-0.000000	0.000000	0.000000	-0.000000	0.000000	-0.050645	0.000000	-0.000000	-0.007945
27.0	-0.000000	0.000000	1.779611	-0.000000	-0.000000	1.779731	0.000000	-0.000000	1.667785
8.0	0.000000	-0.000000	3.407012	0.000000	0.000000	3.446802	-0.000000	0.000000	3.736810

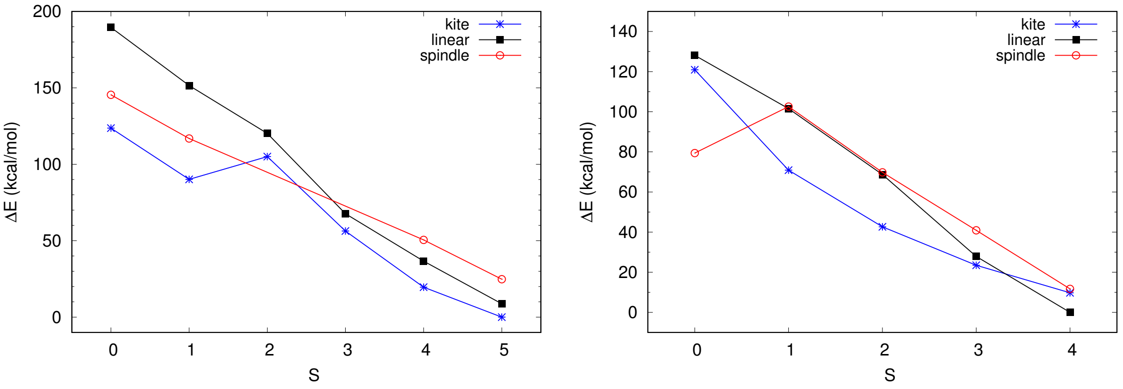

(**a**) Neutral Fe_2O_3

(**b**) Neutral Co_2O_3

Figure S3. RBHHLYP/aug−cc−pVTZ relative energies [kcal/mol] calculated with respect to the lowest-energy states of iron trioxide (left panel) and cobalt trioxide (right panel).

Table S5. Spin contamination for ground state configurations of Fe_2O_3 at the UB3LYP/aug−cc−pVTZ level of theory.

	S	S(S+1)	$\langle S^2 \rangle$
Kite			
Fe_2O_3	4.0	20.00	20.11
$Fe_2O_3^+$	4.5	24.75	25.27
$Fe_2O_3^-$ (ant.)	0.5	0.75	4.63
$Fe_2O_3^-$	4.5	24.75	24.83
Linear			
Fe_2O_3 (ant.)	0.0	0.00	4.97
Fe_2O_3	5.0	30.00	30.05
$Fe_2O_3^+$ (ant.)	0.5	0.75	4.78
$Fe_2O_3^+$	4.5	24.75	24.84
$Fe_2O_3^-$ (ant.)	0.5	0.75	4.62
$Fe_2O_3^-$	4.5	24.75	24.81
Spindle			
Fe_2O_3 (ant.)	0.0	0.00	4.55
Fe_2O_3	5.0	30.00	30.05
$Fe_2O_3^+$ (ant.)	0.5	0.75	5.42
$Fe_2O_3^+$	5.5	35.75	35.79
$Fe_2O_3^-$ (ant.)	0.5	0.75	4.58
$Fe_2O_3^-$	4.5	24.75	24.78

Table S6. Spin contamination for ground state configurations of Fe_2O_3 at the UMP2/aug−cc−pVTZ level of theory.

	S	S(S+1)	$\langle S^2 \rangle$
Kite			
Fe_2O_3 (ant.)	0.0	0.00	5.07
Fe_2O_3	5.0	30.00	30.12
$Fe_2O_3^+$ (ant.)	0.5	0.75	5.78
$Fe_2O_3^-$ (ant.)	0.5	0.75	4.82
Linear			
Fe_2O_3 (ant.)	0.0	0.00	5.13
$Fe_2O_3^+$ (ant.)	0.5	0.75	5.87
$Fe_2O_3^-$ (ant.)	0.5	0.75	4.86
Spindle			
Fe_2O_3 (ant.)	0.0	0.00	4.99
Fe_2O_3	5.0	30.00	30.06
$Fe_2O_3^+$ (ant.)	0.5	0.75	5.88
$Fe_2O_3^-$ (ant.)	0.5	0.75	4.80

Table S7. Spin contamination for ground state configurations of Co_2O_3 at the UB3LYP/aug−cc−pVTZ level of theory.

	S	S(S+1)	$\langle S^2 \rangle$
Kite			
Co_2O_3	3.0	12.00	12.05
$Co_2O_3^+$	3.5	15.75	15.82
$Co_2O_3^-$	3.5	15.75	15.79
Linear			
Co_2O_3 (ant.)	0.0	0.00	3.95
Co_2O_3	4.0	20.00	20.04
$Co_2O_3^+$	3.5	15.75	16.33
$Co_2O_3^-$	3.5	15.75	15.85
Spindle			
Co_2O_3	4.0	20.00	20.05
$Co_2O_3^+$	4.5	24.75	24.79
$Co_2O_3^-$ (ant.)	0.5	0.75	3.56
$Co_2O_3^-$	3.5	15.75	15.78

Table S8. Spin contamination for ground state configurations of Co_2O_3 at the UMP2/aug−cc−pVTZ level of theory.

	S	S(S+1)	$\langle S^2 \rangle$
Kite			
Co_2O_3 (ant.)	0.0	0.00	4.08
Co_2O_3	4.0	20.00	20.06
$Co_2O_3^+$	0.5	0.75	4.43
$Co_2O_3^-$	0.5	0.75	4.81
Linear			
Co_2O_3 (ant.)	0.0	0.00	4.20
Co_2O_3	4.0	20.00	20.22
$Co_2O_3^+$	1.5	3.75	6.89
$Co_2O_3^-$	0.5	0.75	3.91
Spindle			
Co_2O_3	4.0	20.00	20.24
$Co_2O_3^+$ (ant.)	0.5	0.75	4.76
$Co_2O_3^-$ (ant.)	0.5	0.75	3.80

Table S9. Normal vibration frequencies and IR intensities of equilibrium states of Fe_2O_3 found at the UB3LYP/aug−cc−pVTZ level of theory. Freqencies are given in cm^{-1} and IR intensities in $Debye^2/amu\text{-}Angstrom^2$ units.

	Fe_2O_3			$Fe_2O_3{}^-$			$Fe_2O_3{}^+$		
Mode	Freq.	Symm.	IR intensity	Freq.	Symm.	IR intensity	Freq.	Symm.	IR intensity
Kite									
1	116.7799	A″	0.000000	98.6492	B_1	0.265005	57.0150	B_1	0.128875
2	118.5876	A″	0.000000	148.8820	B_2	0.332408	82.4713	B_2	0.097216
3	324.4670	A′	0.678218	197.4372	B_1	1.662205	239.5085	B_1	1.529362
4	324.9196	A′	0.676999	254.3269	A_1	0.006398	288.5959	A_1	0.006604
5	393.3356	A′	0.000002	331.9869	B_2	0.800288	446.2421	B_2	0.009689
6	482.1644	A″	2.255798	461.4775	A_1	3.454928	512.9906	A_1	2.978345
7	608.9272	A′	3.126103	671.9033	B_2	3.814341	655.7119	B_2	1.009801
8	609.2772	A′	3.126485	696.3587	A_1	1.413193	735.6086	A_1	3.189028
9	718.4787	A′	0.000019	831.7190	A_1	5.962178	760.3363	A_1	0.110468
Spindle									
1	116.7799	A″	0.000000	135.6515	A	0.000092	105.8745	A″	0.104975
2	118.5876	A″	0.000000	135.9341	A	0.000088	106.4459	A′	0.105118
3	324.4670	A′	0.678218	294.2027	A	0.783675	349.6466	A′	0.000000
4	324.9196	A′	0.676999	294.9655	A	0.782022	371.3064	A″	0.000000
5	393.3356	A′	0.000002	347.5993	A	1.360901	371.5042	A′	0.000002
6	482.1644	A″	2.255798	453.2782	A	0.000040	543.5428	A′	0.021249
7	608.9272	A′	3.126103	587.7454	A	4.545985	543.5553	A″	0.021281
8	609.2772	A′	3.126485	588.0000	A	4.542790	562.3195	A′	2.834552
9	718.4787	A′	0.000019	670.2523	A	0.000062	693.5481	A′	0.000002
Linear									
1	24.5580	A	0.760606	44.4161	A	0.511535	42.9530	A	0.228056
2	42.4942	A	0.453187	52.1046	A	0.055180	47.6073	A	0.184505
3	90.5044	A	0.000000	113.6801	A	0.019962	89.0130	A	0.248372
4	158.5546	A	1.742584	186.9770	A	1.140799	183.1809	A	1.217314
5	204.8298	A	1.444445	203.7185	A	1.151441	211.3113	A	1.006464
6	315.2391	A	0.000000	276.1401	A	0.360517	296.2377	A	0.145011
7	856.0004	A	0.681215	787.4680	A	6.496292	654.9903	A	1.046794
8	896.9702	A	0.000000	852.5547	A	8.043393	842.8368	A	0.053037
9	937.8494	A	19.064046	868.8987	A	0.290110	943.1704	A	5.489030

Table S10. Normal vibration frequencies and IR intensities of equilibrium states of Co_2O_3 found at the UB3LYP/aug−cc−pVTZ level of theory. Freqencies are given in cm^{-1} and IR intensities in Debye^2/amu-Angstrom^2 units.

	Co_2O_3			$Co_2O_3^-$			$Co_2O_3^+$		
Mode	Freq.	Symm.	IR intensity	Freq.	Symm.	IR intensity	Freq.	Symm.	IR intensity
Kite									
1	69.7444	B2	0.024418	81.2485	B1	0.02104	90.5088	A	0.036363
2	105.8634	B1	0.015939	132.0921	B2	0.45335	144.1934	A	0.276010
3	264.6639	B1	1.079077	177.5060	B1	1.60984	190.5733	A	1.131369
4	265.3706	A1	0.279600	232.9378	A1	0.13694	217.7512	A	0.136971
5	460.4252	B2	0.006241	303.5621	B2	0.62056	474.8202	A	0.276791
6	587.3860	A1	1.661037	412.9970	A1	2.79605	532.4143	A	0.839023
7	667.7424	B2	1.764309	686.1629	B2	2.39977	614.1222	A	1.786425
8	683.0907	A1	0.046850	701.1042	A1	1.08746	673.5287	A	0.325431
9	945.9050	A1	2.878258	833.6508	A1	3.72319	894.5127	A	0.137678
Spindle									
1	112.8177	A	0.060199	21.3133	A	0.30436	144.3644	A	0.6147
2	114.1740	A	0.058391	79.5200	A	0.04969	178.5869	A	0.3664
3	253.6592	A	0.113542	96.1758	A	0.00222	180.5434	A	0.3674
4	255.4606	A	0.115956	197.7786	A	1.89577	220.5228	A	0.0000
5	314.4652	A	0.022901	198.8085	A	1.81202	220.8305	A	0.00000237
6	437.9598	A	2.145990	300.6005	A	0.00000	361.5328	A	0.00000237
7	467.5634	A	0.007540	841.3512	A	0.12536	463.7251	A	0.00000710
8	468.6681	A	0.008082	887.1097	A	0.00002	464.7255	A	0.0000166
9	662.7945	A	0.159188	963.8303	A	20.59277	574.8780	A	0.00000710
Linear									
1	40.9317	A	0.332581	77.0686	A	0.090900	65.5414	A	0.212173
2	77.6006	A	0.104764	114.7766	A	0.000007	49.6085	A	0.202085
3	89.5147	A	0.000007	141.4132	A	0.158895	99.1615	A	0.002920
4	190.5623	A	1.638203	154.1101	A	0.979454	192.1797	A	1.349313
5	198.1133	A	1.864375	174.7284	A	0.346627	242.7155	A	0.865572
6	300.5502	A	0.000000	605.6892	A	0.401847	269.8216	A	0.000078
7	841.3273	A	0.128058	627.3927	A	0.918944	768.1549	A	0.160267
8	887.1507	A	0.000000	869.3518	A	8.570238	811.7842	A	0.031701
9	963.7539	A	20.599047	886.9678	A	2.423530	1095.8787	A	14.210209

Table S11. Normal vibration frequencies and IR intensities of equilibrium states of Fe_2O_3 found at the UMP2/aug−cc−pVTZ level of theory. Freqencies are given in cm^{-1} and IR intensities in $Debye^2/amu\text{-}Angstrom^2$ units.

	Fe_2O_3			$Fe_2O_3^-$			$Fe_2O_3^+$		
Mode	Freq.	Symm.	IR intensity	Freq.	Symm.	IR intensity	Freq.	Symm.	IR intensity
Kite									
1	76.9561	A	0.058211	106.1696	A	0.09963	57.7743	A	0.1982
2	126.5123	A	0.138644	159.7871	A	0.40189	120.1963	A	0.1378
3	231.7914	A	2.216086	202.5208	A	2.32905	252.7303	A	1.7512
4	307.8855	A	0.246208	273.8953	A	0.14338	287.5193	A	0.0308
5	467.6401	A	0.051760	343.8190	A	1.31105	382.5475	A	0.00223
6	609.4270	A	1.914855	473.0888	A	6.66082	598.3358	A	3.1162
7	631.4627	A	2.337503	649.7604	A	1.91602	719.7675	A	3.5039
8	649.1085	A	4.679387	654.0695	A	4.20901	762.5276	A	6.4482
9	723.5427	A	6.714673	868.7625	A	9.98791	788.9826	A	0.4358
Spindle									
1	211.0203	A	0.4048	187.9528	A	0.00129	359.8774	A	0.1885
2	288.9683	A	0.1765	189.2005	A	0.00175	360.7100	A	0.1866
3	290.5693	A	0.3290	306.4143	A	0.5951	390.7267	A	1.1714
4	368.2386	A	0.3279	306.4465	A	0.5927	390.8949	A	1.1760
5	463.6949	A	0.5427	413.8765	A	0.3776	394.3151	A	0.00265
6	480.9973	A	1.5930	445.2858	A	3.1795	621.6895	A	4.6468
7	586.9561	A	3.1396	571.8055	A	6.0671	753.2489	A	0.0597
8	669.4834	A	2.5549	572.1503	A	6.0775	887.4237	A	22.2243
9	779.3629	A	0.5104	671.8852	A	0.0866	888.7186	A	22.3645
Linear									
1	26.7344	A	0.5745	65.0464	A	0.0360	30.6458	A	0.5238
2	54.6409	A	0.5776	55.8737	A	1.0669	62.8923	A	0.0384
3	56.1047	A	0.2376	68.6904	A	1.2989	71.2977	A	0.0226
4	108.5691	A	0.3662	91.4186	A	0.0308	142.4557	A	1.7759
5	119.6508	A	1.7819	98.5344	A	0.0009	207.2493	A	1.6884
6	294.9701	A	0.0138	296.9097	A	0.088	272.5702	A	0.2135
7	635.2737	A	1.4531	829.3242	A	1.2403	685.7041	A	3.9705
8	873.7115	A	11.3932	898.2614	A	6.7174	732.9376	A	0.0196
9	949.8769	A	13.8571	920.3455	A	33.611	982.1661	A	20.132

Table S12. Normal vibration frequencies and IR intensities of equilibrium states of Co_2O_3 found at the UMP2/aug−cc−pVTZ level of theory. Freqencies are given in cm^{-1} and IR intensities in $Debye^2/amu\text{-}Angstrom^2$ units.

	Co_2O_3			$Co_2O_3^-$			$Co_2O_3^+$		
Mode	Freq.	Symm.	IR intensity	Freq.	Symm.	IR intensity	Freq.	Symm.	IR intensity
Kite									
1	31.2551	A	0.070	92.8003	A	0.047	70.9240	A	0.001
2	108.3613	A	0.094	144.6395	A	0.420	182.1121	A	0.308
3	114.1342	A	0.126	205.4517	A	0.082	266.6469	A	0.028
4	159.4178	A	1.820	294.7237	A	1.130	367.8398	A	0.933
5	168.9634	A	2.207	391.9299	A	0.837	701.1298	A	11.72
6	286.4910	A	0.0000	551.2736	A	7.446	718.8813	A	9.57
7	601.5732	A	0.456	573.1637	A	7.780	767.6353	A	28.56
8	645.9927	A	0.00001	584.0284	A	3.197	839.6502	A	2.07
9	947.4080	A	15.5600	868.8759	A	3.750	994.8517	A	18.10
Spindle									
1	202.3205	A	0.300	176.7974	A	1.180	103.6019	A	0.666
2	209.5869	A	1.169	101.0273	A	2.484	178.6989	A	0.762
3	227.0963	A	0.449	206.2186	A	0.074	251.2253	A	0.192
4	285.2885	A	0.502	321.3108	A	0.402	275.1591	A	0.032
5	313.1089	A	0.066	343.0386	A	3.380	328.1253	A	0.032
6	430.3000	A	1.052	396.2924	A	0.407	414.5447	A	3.580
7	477.7307	A	1.543	571.0084	A	7.536	452.0901	A	1.411
8	507.7256	A	3.561	587.4573	A	2.457	494.4864	A	1.427
9	647.6072	A	2.121	641.4474	A	0.198	539.3713	A	0.011
Linear									
1	34.66	A	0.034	22.16	A	0.242	43.33	A	0.040
2	98.49	A	0.000	114.50	A	0.217	93.50	A	0.063
3	98.49	A	0.000	114.50	A	0.217	93.50	A	0.063
4	151.32	A	1.926	188.91	A	1.519	169.55	A	0.718
5	151.32	A	1.926	188.91	A	1.519	169.55	A	0.718
6	288.03	A	0.000	274.88	A	0.042	242.05	A	0.182
7	740.15	A	11.243	814.40	A	2.291	357.88	A	0.020
8	810.85	A	0.000	867.39	A	9.729	766.64	A	0.614
9	953.08	A	19.495	941.35	A	26.014	969.32	A	7.087

Table S13. RMP2/aug−cc−pVTZ and RB3LYP/aug-c-pVTZ relative energies of states of FeO molecule.

	MP2/aug−cc−pVTZ		RB3LYP/aug−cc−pVTZ	
Spin	Energy [Hartree]	Rel. energy [kcal/mol]	Energy [Hartree]	Rel. energy [kcal/mol]
0	-1337.6474561995	57.95	-1338.7404630869	64.95
1	-1337.6814377671	36.63	-1338.7627997278	50.93
2	-1337.7398044550	0.00	-1338.8439678196	0.00
3	-1337.6907044969	30.82	-1338.8085711773	22.21

Table S14. RMP2/aug−cc−pVTZ and RB3LYP/aug-c-pVTZ relative energies of states of FeO^-.

	MP2/aug−cc−pVTZ		RB3LYP/aug−cc−pVTZ	
Spin	Energy [Hartree]	Rel. energy [kcal/mol]	Energy [Hartree]	Rel. energy [kcal/mol]
1/2	-1337.6626911230	83.87	-1338.8190961386	43.68
3/2	-1337.7906623468	3.57	-1338.8884560178	0.16
5/2	-1337.7963452917	0.00	-1338.8887072567	0.00
7/2	-1337.6988878906	61.15	-1338.8007357281	55.20

Data from antiferromagnetic singlet computations

October 22, 2025

1 Fe_2O_3 kite

Step 1 - We optimize the structure for the high-spin state (S=5)

```
----------------------------------------------------------------------
#p UB3LYP/aug-cc-pVTZ Freq Opt=(MaxCycles=2000,VeryTight) SCF=(Vshift=
 1,Intrep,VeryTight,NoVarAcc,CDIIS,MaxCycle=2000,Save) IOp(1/6=500,5/7=
 800,5/85=800,5/13=1,5/36=1,8/11=1) Use=L506 GFInput
 ----------------------------------------------------------------------
-------------------------------------
 Unrestricted openshell Fe2O3_kite opt
 -------------------------------------
 Charge =  0 Multiplicity =11

         Item               Value     Threshold  Converged?
 Maximum Force            0.000000     0.000002     YES
 RMS     Force            0.000000     0.000001     YES
 Maximum Displacement     0.000002     0.000006     YES
 RMS     Displacement     0.000001     0.000004     YES
 Predicted change in Energy=-5.766233D-13
 Optimization completed.
    -- Stationary point found.

                          Input orientation:
 ---------------------------------------------------------------------
 Center     Atomic      Atomic             Coordinates (Angstroms)
 Number     Number       Type             X           Y           Z
 ---------------------------------------------------------------------
      1         26           0       -0.000000    0.000000   -0.816400
      2         26           0        0.000000   -0.000000    1.824905
      3          8           0       -0.000000    0.000000   -2.477912
      4          8           0       -1.351967   -0.000000    0.734703
      5          8           0        1.351967   -0.000000    0.734703
 ---------------------------------------------------------------------
```

```
 Stoichiometry    Fe2O3(11)
 Framework group  C2V[C2(OFeFe),SGV(O2)]
 Deg. of freedom     4
 Full point group                 C2V     NOp   4
 RotChk:  IX=0 Diff= 6.20D-16
 Largest Abelian subgroup         C2V     NOp   4
 Largest concise Abelian subgroup C2      NOp   2
                        Standard orientation:
 ---------------------------------------------------------------------
 Center     Atomic      Atomic             Coordinates (Angstroms)
 Number     Number       Type             X           Y           Z
 ---------------------------------------------------------------------
      1         26           0       -0.000000   -0.000000   -1.055256
      2         26           0        0.000000    0.000000    1.586048
      3          8           0       -0.000000   -0.000000   -2.716769
      4          8           0        0.000000    1.351967    0.495847
      5          8           0       -0.000000   -1.351967    0.495847
 ---------------------------------------------------------------------

 Mulliken charges and spin densities:
               1          2
     1  Fe   1.020786   4.026327
     2  Fe   1.071706   3.855320
     3  O   -0.644940   0.882626
     4  O   -0.723776   0.617864
     5  O   -0.723776   0.617864

Version=ES64L-G16RevC.02\State=11-A1\HF=-2753.3081581\S2=30.046918\S2-1=0.\S2A=30.000373
```

Step 2 - We check the stability of the high-spin wavefunction in the final geometry of the optimization

```
---------------------------------------------------------
#p UB3LYP/aug-cc-pVTZ guess=read geom=allcheck stable=opt
---------------------------------------------------------
 Charge =  0 Multiplicity =11
 Redundant internal coordinates found in file.

                        Input orientation:
 ---------------------------------------------------------------------
 Center     Atomic      Atomic             Coordinates (Angstroms)
 Number     Number       Type             X           Y           Z
 ---------------------------------------------------------------------
      1         26           0       -0.000000    0.000000   -0.816400
      2         26           0       -0.000000   -0.000000    1.824905
      3          8           0       -0.000000    0.000000   -2.477912
```

```
     4          8           0       -1.351967   -0.000000    0.734703
     5          8           0        1.351967   -0.000000    0.734703
 ---------------------------------------------------------------------

*********************************************************************
 Stability analysis using <AA,BB:AA,BB> singles matrix:
 *********************************************************************
 1PDM for each excited state written to RWF  633
 Ground to excited state transition densities written to RWF  633

 Eigenvectors of the stability matrix:

 Eigenvector   1:  11.010-B1   Eigenvalue= 0.0492174  <S**2>=30.058
     28B -> 38B       -0.15656
     31B -> 42B        0.14998
     32B -> 34B        0.35929
     32B -> 37B        0.18531
     32B -> 38B        0.76600
     33B -> 36B        0.34207
     33B -> 41B       -0.22050
The wavefunction is stable under the perturbations considered.
 Leave Link  914 at Sat May 31 21:48:10 2025
 The wavefunction is already stable.
 Leave Link  508 at Sat May 31 21:48:10 2025
 Copying SCF densities to generalized density rwf, IOpCl= 1 IROHF=0.

Version=ES64L-G16RevC.02\State=11-A1\HF=-2753.3081581\S2=30.046918\S2-1=0.\S2A=30.000373
```

Step 3 - Fragment guess job using the optimized high-spin geometry in Step 1

```
---------------------------------------------
#p UB3LYP/aug-cc-pVTZ guess=(Fragment=5,only)
---------------------------------------------

---------------------------------------
 fragment guess using High-Spin geometry
 ---------------------------------------
 Symbolic Z-matrix:
 Charge =  0 Multiplicity = 1 in supermolecule
 Charge =  3 Multiplicity = 6 in fragment      1.
 Charge =  3 Multiplicity =-6 in fragment      2.
 Charge = -2 Multiplicity = 1 in fragment      3.
 Charge = -2 Multiplicity = 1 in fragment      4.
 Charge = -2 Multiplicity = 1 in fragment      5.
 Fe(Fragment=1)        0.        0.      -0.8164
```

```
 Fe(Fragment=2)        0.         0.         1.82491
 O(Fragment=3)         0.         0.        -2.47791
 O(Fragment=4)        -1.35197   0.         0.7347
 O(Fragment=5)         1.35197   0.         0.7347
```

Step 4 - Stability calculation on the resulting singlet wavefunction from Step 3

```
----------------------------------------------------------------------
#p UB3LYP/aug-cc-pVTZ scf=nosymm guess=read geom=allcheck stable=opt p
 op=nbo7
----------------------------------------------------------------------

Charge =  0 Multiplicity = 1
Redundant internal coordinates found in file.  (old form).

                         Input orientation:
 ---------------------------------------------------------------------
 Center     Atomic      Atomic             Coordinates (Angstroms)
 Number     Number       Type             X           Y           Z
 ---------------------------------------------------------------------
      1         26           0        0.000000    0.000000   -0.816400
      2         26           0       -0.000000   -0.000000    1.824905
      3          8           0        0.000000    0.000000   -2.477912
      4          8           0       -1.351967   -0.000000    0.734703
      5          8           0        1.351967   -0.000000    0.734703
 ---------------------------------------------------------------------

SCF Done:  E(UB3LYP) =  -2753.31211398     A.U. after   26 cycles
           NFock= 26  Conv=0.44D-08     -V/T= 2.0017
 <Sx>= 0.0000 <Sy>= 0.0000 <Sz>= 0.0000 <S**2>= 4.9045 S= 1.7704

 Mulliken charges and spin densities:
              1          2
     1  Fe   1.025572   3.974596
     2  Fe   1.072899  -3.822968
     3  O   -0.640830   0.882762
     4  O   -0.728820  -0.517195
     5  O   -0.728820  -0.517195

\Version=ES64L-G16RevC.02\State=1-A1\HF=-2753.312114\S2=4.904527\S2-1=0.\S2A=15.224294
```
